# Psychosocial impacts of post-disaster compensation processes: narrative systematic review

**DOI:** 10.1186/s40359-024-02025-9

**Published:** 2024-10-07

**Authors:** Samantha K. Brooks, M. Brooke Rogers, Simon Wessely, Sonny S. Patel, Neil Greenberg

**Affiliations:** 1 Department of Psychological Medicine, Weston Education Centre, King’s College London, London, SE5 9RJ UK; 2 Department of War Studies, King’s College London, The Strand, London, WC2R 2LS UK; 3 Transcultural Conflict and Violence Initiative, Georgia State University, Atlanta, GA USA; 4 Department of Global Health and Population, Harvard T.H. Chan School of Public Health, Boston, MA USA

**Keywords:** Communities, Compensation, Disasters, Mental health, Systematic review, Wellbeing

## Abstract

After disasters, many people seek compensation for physical, psychological or economic damages. However, compensation processes can be perceived as arduous and unfair and potentially create stress for both individuals and communities. This systematic review explored the psychosocial impacts of post-disaster compensation processes, including compensation sought through both litigation and government assistance programmes. We searched seven databases, hand-searched reference lists of included studies, and used thematic analysis to synthesise results of included studies. We screened 6,532 papers, ultimately including 66 in the review. While we found mixed evidence regarding the relationship between individual mental health and the compensation process, many studies suggested the process placed demands on emotional resources and could cause stress. Numerous challenges of the compensation process were described, including complicated paperwork, lengthy processes, inadequate information, confusing eligibility criteria, lack of inter-agency cooperation, poor understanding of communities’ unique needs, insufficient pay-outs, and politicisation of the process. Inequities in compensation distribution introduced additional stress to already traumatised communities, who often experienced resentment, envy and conflict. The mixed nature of the relationship between mental health and the compensation process was evident in research trends where a small number of studies reported positive findings related to relating to gratitude, helpfulness of compensation and strengthened community relationships, while a substantial number of others reported negative impacts including higher mental health problems. Positive and negative impacts were reported for both litigation and non-litigation compensation-seeking. The nuanced dynamics of these findings are described in greater detail within the paper. It is important that compensation regulators consider the potential impacts on individuals and communities and take steps to address compensation inequities. This enhanced understanding of how those affected by disasters can rebuild their lives and furthering understanding of how to support them will enable evidence-based approaches to building resilience and planning for long-term recovery. Significant compensation process improvements could be realised by ensuring clear communication and transparent decision-making. Overall, this review underscores the importance of ensuring that compensation processes are fair and straightforward so they can repair material losses without deteriorating the social norms and relationships of affected communities.

**Supplementary Information:**

The online version contains supplementary material available at 10.1186/s40359-024-02025-9.

## Introduction

Globally, disasters and extreme events – including those caused by natural hazards and responsible party disasters – are increasing [1–6]. The last century has seen a huge increase in post-disaster compensation-seeking (including disaster assistance, compensation sought through litigation, and compensation funds set up to financially assist survivors without litigation).

Outside of the disaster context, heated debates about individual compensation and secondary gain have lost and gained traction over the last century. Illness following trauma first became an acceptable reason for seeking compensation in the late nineteenth century [7–9]. At the time, little was understood about the potential long-term effects of traumatic injuries so diagnoses were often associated with accusations of fraudulent claims for compensation [10]. Similarly, after World War I it was suggested that many people intentionally exaggerated or fabricated symptoms for personal gain [11], causing many pension claimants anxiety about being accused of malingering [12] and the idea of seeking compensation for psychological injuries was viewed negatively [13]. Until late in the twentieth century when post-traumatic stress disorder (PTSD) became better understood, post-traumatic symptoms were often attributed to desire for *secondary gain* – the benefits one might gain as a result of failing to recover [14–17]. Although evidence to the contrary was provided [18, 19], claiming compensation for psychological damage continued to be controversial throughout the twentieth century, with many suggesting that compensation provided an incentive for exaggerating symptoms or remaining ill [20]. However, most recent research suggests there is little evidence that symptoms are exaggerated for secondary gain [21, 22].

Questions remain as to whether compensation processes themselves can impact mental health; there is a wealth of literature on the psychological impact of compensation processes in non-disaster contexts but the issue remains contentious [23]. A number of studies report higher mental health problems in people involved in the compensation process than those not involved; a meta-analysis of the impact of compensation processes outside of the disaster context found that this was true for both those involved in litigation and those seeking other types of compensation, including worker’s compensation and no fault compensation processes [23]. However, this finding alone is not evidence that the compensation process exacerbates symptoms; those with more symptoms may be more likely to seek compensation in the first place [24]. Additionally, litigants could develop a ‘compensation mindset’ due to having to portray themselves as distressed at assessments [23]. Recent years have seen greater focus on the elements of the compensation process which might lead to worse psychological outcomes, such as perceptions of informational and interpersonal justice [25] or rejected claims [26].

### Post-disaster compensation

Disasters can have profound effects on individuals and communities. Mental health problems such as PTSD, depression and anxiety disorders are frequently experienced [27], exacerbated by property damage and financial struggles [28, 29]. Man-made disasters such as nuclear accidents may involve particularly severe impacts through the process of ‘risk amplification’ [30–32]. Economic situations might be negatively affected due to direct costs of medical treatment and indirect costs such as losses of earning capacity, livelihood or property. Unsurprisingly, issues relating to money are key stressors after extreme events. Money is central to our lives [33] and many of our most fundamental needs [34] are strongly related to money (e.g. food, shelter, employment, resources and property), all of which can be compromised after a disaster.

After a disaster, there may be money provided to ‘level the playing field’, to ensure that people are not financially challenged by the disaster and restore the status quo ante; recent examples of this are COVID-19 payments [35]. There may also be money which actively seeks to give monetary recompense for pain, suffering, injury, or loss of future prospects – this is often decided by personal injury litigation, but states can step in to provide victim compensation schemes in which payment is made as a recompense for suffering [36]. In the aftermath of a disaster, survivors often seek compensation in the form of financial and material assistance to help recover losses or in the form of monetary awards to ‘compensate’ for harm done. After disasters caused by natural hazards, compensation often comes from privately-held insurance, government assistance programmes or non-profit organisations, where the process might involve filing claims through facilities set up to compensate for material losses or physical harm [1, 36]. After a disaster where there is perceived to be a ‘responsible party’, the quest for compensation typically falls to tort law [1] with litigation allowing the injured parties to recover damages in court for losses, injuries or psychological damage. There is a third type of compensation process: ‘responsible parties’ might establish a compensation fund with the aim of distributing funds quickly as well as discouraging people from pursuing litigation [36, 37]. Local governments may also provide victim compensation as an alternative to tort recovery, for altruistic reasons, or when the government should have been able to prevent the disaster [38, 39]. In other words, there are three main ways of being ‘compensated’ after a disaster: filing claims through facilities set up to compensate for material loss or physical harm; tort law; and compensation from ‘responsible parties’ (which may sometimes include local governments if the disaster should have been preventable).

In the case of ‘responsible party’ disasters there is often a desire for justice driving individuals to seek compensation. Literature on victims of injustice defines ‘compensatory justice’ as the provision of resources with the goal of reversing or minimising the impact of harm [40]. In the disaster context, though, compensation may not ‘reverse the impact’ or return an individual to their pre-disaster state but may instead repair individuals’ capacity to flourish [41]. Aspects of justice are intertwined with the compensation process itself – not only for litigants, but for those who seek disaster assistance or awards from compensation funds. For example, the process involves distributive justice (fair distribution of resources), procedural justice (fair decision-making processes to evaluate claims), interpersonal justice (fair treatment during the process) and informational justice (provision of adequate, timely information) [37, 42, 43].

However, post-disaster compensation processes can inadvertently introduce new tensions and problems if not handled with care [44] and can contribute to post-disaster anxiety and depression [45], deplete time, money and energy [46] and potentially lead to stress [47]. For those who have to go through litigation in order to gain compensation, the experience can lead to anxious and depressive symptoms directly relating to the litigation [48] as most litigants have little understanding of court procedures or appreciation of the adversarial nature of litigation [49]. Survivors risk experiencing a second victimisation as they pursue legal battles for compensation [50], which can be extremely complex and lengthy [51, 52].

Additionally, the compensation process is often perceived as unfair or unjust. The concept of *fairness* is a fundamental part of human life [53–55]. In the context of disaster-related compensation-seeking, where the amount of compensation received (if any) is typically dependent on individual circumstances, social comparisons are inevitable [56] which can result in feelings of relative deprivation and foster envy, bitterness, anger and resentment [44, 57, 58]. This can harm the social capital that is vital for collective recovery [59]. In an article about the uneven response to compensation claims following the Hillsborough disaster in the United Kingdom, it was declared that *“the law is plainly a lottery in which some bystanders get splattered with blood and others with money”* [60, p.429]. Across the world are similar stories of perceived injustices within the post-disaster compensation process [61–63]. For example, the World Trade Center (WTC) disaster saw an unprecedented amount of both litigation and compensation paid out via the 9/11 Fund [64]; in the aftermath of the 7/7 London bombings, attention was drawn to the disparity between the compensation provided to the families of those killed in the London attacks to the much higher pay-outs received by relatives of the 9/11 terror attacks [65, 66].

The value of strong social networks after a disaster has been well-established [67, 68]. However, unfairness in compensation pay-outs could undermine this. For example, Freudenburg and Jones [69] described ‘corrosive communities’ emerging in the aftermath of technological disasters, characterised by anger, isolation and loss of trust in others. Worryingly, there is also evidence that compensation disparities may reflect structural and systemic inequities; processes of bias and discrimination as well as differences in access and power could disadvantage minorities and already-vulnerable groups [70–73]. For example, a study examining factors associated with approval of insurance claim pay-outs and assistance grants following Hurricane Sandy in the USA found a significant negative association between assistance approval rate and ‘minority’ ethnicity (that is, African American or Hispanic as opposed to White) [71]. A study tracking wealth trajectories in the US after natural hazards [72] found that Black and Latino residents were more likely than White residents to lose wealth following a disaster, as were residents with lower levels of education and those who rented their homes rather than owned them. It is also the case that that the poorest disaster survivors may have the most difficulties in accessing both legal services and compensation funds. Hooks and Miller [73] describe how the rules regarding how to apply for monetary assistance are ill-designed to meet the needs of low-income families: after Hurricane Katrina, procedures and policies around applying for assistance limited the availability of assistance by eliminating paper applications, meaning those without access to a phone or the internet were unable to apply.

### Aims

There is a dearth of empirical research on post-disaster compensation claims [74] but it is important to understand the potential impacts as the need for post-disaster compensation is increasing [75, 76]. As the topic has not yet been the subject of a systematic review, we aimed to fill this gap by exploring whether there are psychological or social impacts of post-disaster compensation processes and, if so, which aspects of the process affect outcomes.

## Method

We conducted a systematic review following the Preferred Reporting Items for Systematic Reviews and Meta-analyses (PRISMA) guidelines [77]. The PRISMA checklist is provided as Appendix 1. Based on the PICO framework [78] (although we did not require a comparator group), we defined our research question as: In disaster-affected individuals and communities, do post-disaster compensation processes affect individual and/or community wellbeing?

### Defining key terms

Before describing methodology, it is worth defining some key terms. Firstly, by *disaster*, we mean a hazardous event which results in serious disruption to the functioning of a community or society due to human, material, economic or environmental losses and impacts [79].

By *compensation*, we refer to compensation through any means: disaster assistance, litigation, or compensation funds set up specifically to avoid the litigation process. Dixon & Kaganoff Stern [37] note that while *compensation* and *assistance* are distinct forms of financial assistance, the distinction between the terms is blurred: while disaster assistance refers to payments intended to help individuals or businesses get back on their feet and restore stability, this assistance does also partly provide compensation for some of the losses experienced. Throughout this review, we use *compensation* as a catch-all term incorporating disaster assistance, compensation funds, and litigation; in the Results section, we differentiate between litigation and non-litigation compensation where necessary to do so – for example, where particular findings are based *only* on litigation or non-litigation studies rather than a combination, and therefore cannot be generalised. The term *compensation* throughout this review therefore does not lay claim to the *intent* of the money, nor whether the recipients were ‘made whole’ by it [37].

Additionally, it is worth clarifying what we mean by *mental health* which is referred to throughout the manuscript. We opted to take a broad approach to this in order to capture all potential psychological outcomes. Therefore, we considered both mental health *disorders* (e.g. depression, PTSD, anxiety) and *wellbeing* more generally (e.g. stress, resilience, coping).

### Registering the review

A protocol for this review was prepared and the review was registered on PROSPERO on March 10th 2023 [Registration CRD42023406038]. The only amendment to the information provided at registration was that, after trialling the search strategy and screening a sample of the results, we noted that the term ‘compensation’ is often used interchangeably within the literature to describe litigation, disaster assistance and various compensation schemes. Therefore, although we initially intended to focus on legal claims, we expanded the review to cover different types of compensation.

### Search strategy

We incorporated Patient and Public Involvement and Engagement (PPIE) into the design of the review by sharing proposed search strategies with several lawyers known to the last author, who reviewed our proposed search terms and suggested additional terms based on their knowledge and experience. The final search strategy encompassed three search strings. The first included terms relating to the compensation process; the second included disaster-related terms; and the third included terms relating to either individual or community wellbeing. Search terms within each string were combined using the Boolean operator “OR” and the three strings were then combined together using “AND”. The full search strategy is presented in Appendix 2.

On 11th March 2023, the first author entered the search strategy into seven electronic databases: Medline, Embase, PsycInfo, Global Health, Social Policy and Practice, Health Management Information Consortium, and Web of Science. There were no limitations relating to date, publication type or study type, but studies were restricted to those published in the English language. Database searches were supplemented by hand-searches of reference lists of included papers.

### Data screening

All citations were downloaded to EndNote version X9 (Thomson Reuters, New York, NY) and screened to confirm their relevance to the review against pre-specified inclusion criteria (see Appendix 3). The first author screened 100% of the citations, first by title (excluding any which clearly were not relevant to the review), then by abstract (again, excluding any which clearly were not relevant) and finally by screening full texts, using the selection criteria to determine whether or not each study warranted inclusion. The fourth author also screened the first 10% of the citations independently. Any discrepancies in inclusion/exclusion between the two authors were discussed with the last author.

### Data extraction

Data from the included studies were extracted systematically by the first author into Microsoft Excel. Extracted data included: study authors, publication year, country, design, funding, disaster type, number of participants, age and gender of participants, sampling method, time-point(s) of data collection, compensation claim status, psychological or psychosocial wellbeing outcomes, outcomes relating to perceptions of the compensation process. The fourth author carried out data extraction in duplicate, using the first 10% of included studies. Due to 100% consensus between the two authors across this 10% of studies, no further duplicate screening was carried out.

### Quality appraisal

Quality of quantitative studies was assessed by the first author using a slightly modified version of the AXIS tool [80], consisting of twenty questions assessing studies on various aspects of their research design, analysis and conclusions. We modified two questions so that a ‘yes’ response would indicate higher quality, consistent with the other items, as has been done in previous reviews [81]. Quality of qualitative studies was assessed by the first author using a slightly modified version of the Critical Appraisal Skills Programme (CASP) Qualitative Checklist [82], with one question reworded so that each question was answered with a yes/no response. For mixed-methods studies, either the AXIS or CASP tool was used depending whether quantitative or qualitative data were more prominent in the study. One included study did not report the methodological design used and so this study did not undergo quality appraisal and was automatically labelled ‘poor quality’.

Although there are no standardised cut-off scores for the AXIS or CASP tools, we wanted to be able to categorise studies as high or low quality. A systematic review of different quality assessment tools [83] found only one review [84] used cut-off scores in their quality appraisal. Based on their cut-off percentages, we classed studies as ‘poor quality’ if they scored under 30%; ‘moderate quality’ if they scored 30-60%; ‘good quality’ if they scored 61-80%; and ‘very high quality’ if they scored 81%+.

### Data synthesis

Data were narratively synthesised. The first and second authors worked collaboratively to group results together by theme by carrying out analysis of the extracted data informed by Braun and Clarke’s thematic analysis framework [85]. For example, all data relating to individual mental health/wellbeing impacts were initially coded ‘mental health’ and grouped together in one theme, which was later renamed *Impact of compensation processes on individual mental health and wellbeing* as this better reflected and summarised the data.

## Results

**Fig. 1:**
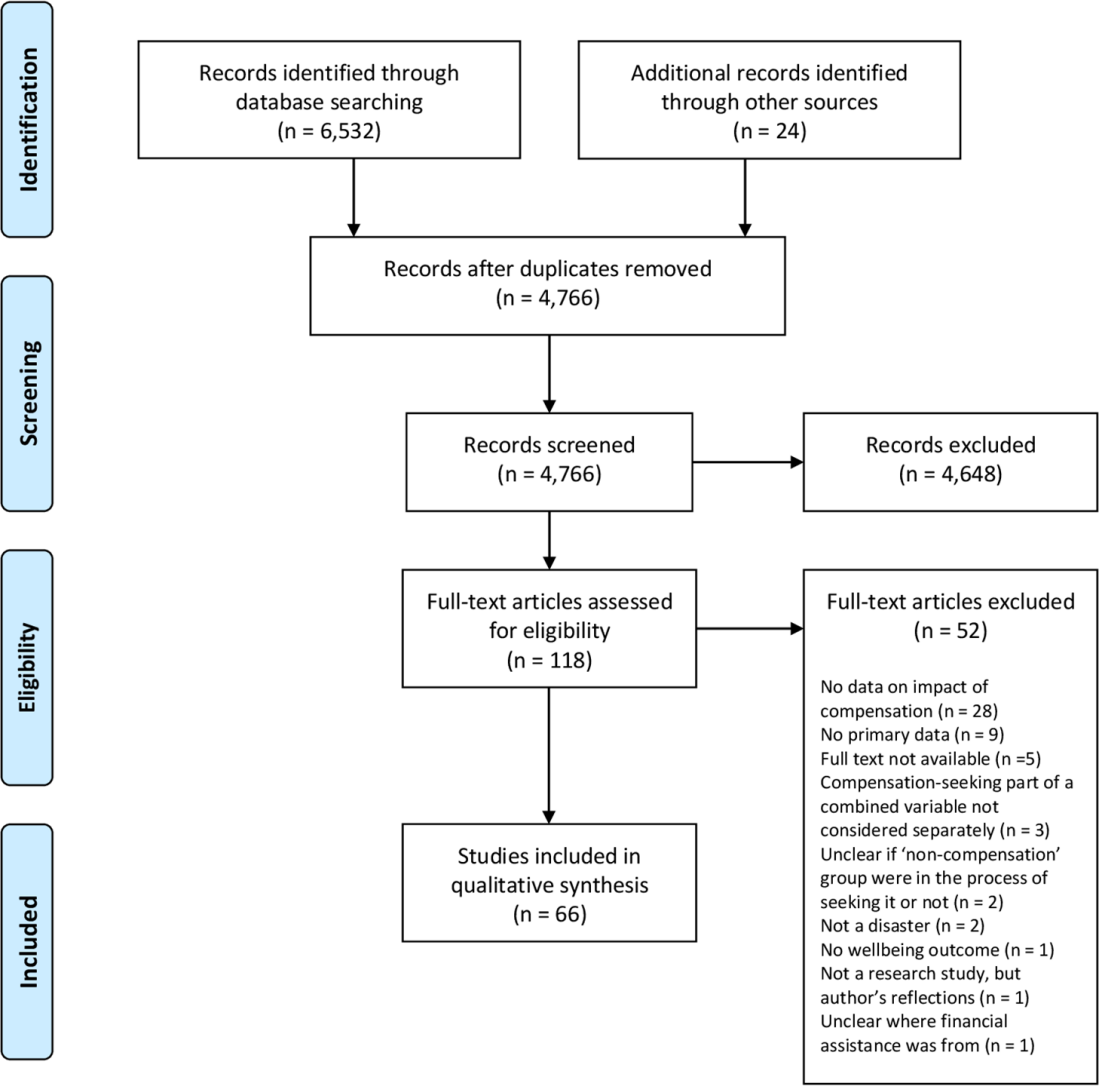
PRISMA 2009 flow diagram

A total of 6,532 citations were found via database searches. Citations were imported into EndNote where 1,790 duplicates were removed. Title screening resulted in the exclusion of 4,190 studies and abstract screening excluded a further 458. Five full texts were unavailable and 47 were excluded for not meeting all inclusion criteria, leaving 42 studies for inclusion. Hand-searches of the reference lists of included studies located an additional 24 studies, resulting in a total of 66 studies for review [23, 36, 37, 46, 68, 74, 86–145]. A flow diagram of the screening process is presented in Fig. 1.

A summary of study characteristics is presented in Additional Table 1: Characteristics of included studies and further details on the compensation processes involved in each study are presented in Additional Table 2: Compensation process details of included studies. Studies came from various countries across North America, Europe, Asia and Oceania. They focused on a fairly even split of disasters caused by natural hazards and manmade disasters. Of those reporting sample size, study populations ranged from 12 to 2,862. The majority reported on local/governmental compensation schemes or disaster assistance, while 24/66 studies reported on the effects of litigation.

The quality of included studies was fair overall, with almost half (32/66) rated as good or very high quality. Only five studies were rated as ‘poor quality’. We generally found similar findings emerging from the literature regardless of quality; findings specific to very high-quality studies are pointed out within the analysis. Quantitative studies tended to lose points for failing to justify sample sizes, describe the representativeness of the sample or provide details of non-responders. Qualitative studies tended to lose points for failing to consider the researchers’ own role and potential biases during data collection/analysis. Many studies also lost points for failing to report on ethics; only 12/66 (18%) described ethical considerations or having the approval of an ethics board.

Thematic analysis resulted in the identification of a typology of compensation impacts, presented in Additional Table 3: A typology of compensation impacts on mental health and individual/community wellbeing.

### Impact of compensation processes on individual mental health and wellbeing

Additional Table 3 summarises the results of our thematic analysis of compensation-related impacts on mental health and wellbeing across two key areas: (1) mental health and wellbeing impacts related to time, emotions, and experience of the compensation process; and (2) procedural (in)justices (procedural, interpersonal, and distributive). Procedural (in)justices are framed as such as they have the potential to be perceived or experienced as positive or negative by individuals and communities. Almost 40% of the papers demonstrated that systemic challenges within compensation processes were associated with a wide variety of primarily negative impacts on individual mental health and wellbeing. Almost half of the studies highlighted procedural (in)justices underpinning individual and community level health and wellbeing impacts related to compensation systems. This is offset by a little over 20% of reports noting positive impacts.

While this visual representation may indicate clear distinctions and relationships between the different aspects of compensation systems and impacts, many of the impacts fall into more than one category within the typology. Several of the stressors interact across time, while others may combine to increase the risk of people experiencing negative mental health and wellbeing impacts. None of the studies examined inter-relationships between the various stressors and their cumulative impact on mental health and wellbeing. Trends and nuances are set out below. Differences between compensation types are noted where evidence supports this differentiation. First, it is important to reflect upon statistical associations in light of the variety of methodological approaches, populations, compensation systems, and disasters noted above.

### Mental health and wellbeing: Statistical associations

A small number of the included quantitative studies assessed statistical associations between mental health outcomes and seeking compensation through litigation, with mixed findings. Some found that litigation status significantly predicted mental health outcomes such as abnormal mood, intrusive stress, avoidant stress, depression and PTSD [74, 86–90]. Another [23] found no differences between litigants and non-litigants in terms of anxiety, depression, functioning or psychiatric symptoms although litigants did have significantly higher rates of generalised anxiety disorder. One study [91] found that having an insurance claim pending or denied was significantly associated with mental health in bivariate analyses but no longer significant after controlling for other variables. Tsujiuchi [92] found no compensation-related difference in stress levels in the first three years post-disaster, but in the fourth year, post-traumatic stress symptoms were significantly higher in those who received compensation because they could never return to their homes and those who received almost no compensation.

Several quantitative studies, both of litigation and other compensation processes, explored the association between mental health and outcomes or perceptions of the claims process. Stress resulting from time spent with lawyers, recurrent unpleasant memories of the disaster and struggling to understand the complex litigation process had a significant direct effect on intrusive symptoms [93]. Finding the compensation process as disturbing as the disaster significantly predicted intrusive and avoidance-related stress [74, 94]. Finding the process too long and a source of community disruption also predicted avoidance behaviours [94].

Quantitative studies not related to litigation also explored the effect of the compensation claims process. Failing to receive compensation for damage significantly predicted depression and suicidal ideation [95]. Unresolved compensation issues significantly predicted PTSD [96]. Satisfaction with the compensation process predicted better physical and mental health [97] whereas dissatisfaction predicted poorer social psychological wellbeing in one study rated as very high-quality [98].

### Mental health and wellbeing trends

The first theme identified in Additional Table 3 generates insight into the mental health and wellbeing impacts of compensation processes over time, identifies a diversity of emotions experienced when engaging with compensation processes, and highlights the most challenging and impactful aspects of compensation processes.

First, few studies carried out longitudinal research to examine the long-term impacts of the compensation process. The majority of those that did focused on litigation after manmade disasters. A study of the effects of the 1989 Exxon Valdez oil spill [99] reported that the mean differences between litigants’ and non-litigants’ stress increased over time, suggesting post-disaster recovery was impeded by the litigation process. The same population was studied by Picou [86] who found that stress levels for litigants were high while the litigation process was ongoing, and at their highest in 2006 when the punitive damage award was cut by half.

Similarly, one mixed-methods study [100] of data collected over 13 years found that being a litigant consistently predicted intrusive stress, and that stress began to decline after the distribution of damage awards. A quantitative study [101] noted that PTSD symptoms were higher following the result of court hearings which did not meet the expectations of the victims. A mixed-methods study [102] found that PTSD could persist for at least three years post-disaster, whereas a quantitative study [103] found that perceptions of injustice in the compensation process predicted stress over the first year after the disaster but not stress three years later.

Second, while the majority of longitudinal studies noted above focused on the experience of litigation processes, qualitative data identified a diverse range of negative impacts of both litigation and other compensation processes on mental health and wellbeing. Respondents reported stress as a recurring theme linked to initiating the process of determining liability [105], as well as disputes with various agencies involved in the pay-out process [104], Compensation processes were described as stressful [46, 74] and re-traumatising [106]. In terms of emotions, participants taking part in these stressful engagements described disappointment [107], anger [108], exhaustion [106], feeling powerless [46, 106], feeling defeated [106], humiliation [105], distress [108], pessimism [106], stress [105, 109] and feeling worried [46], with the claims process perceived as having placed substantial demands on emotional resources [46, 106]. Symptoms such as sleep problems and physical health complaints were attributed to the compensation process by participants in one study rated as very high-quality [46]. Participants in both litigation and non-litigation studies described the claims process being as distressing as, or more distressing than, the disaster itself [46, 74, 106]. In contrast, one study [109] saw respondents label the process of seeking compensation as a means of coping by providing a distraction and the opportunity to do something meaningful in the aftermath. However, most participants reported that the process became a source of stress over time.

Third, participants across studies cited time-consuming and complicated paperwork and the length of the process as significant variables informing their experience of and impact of the processes upon them. For example, many studies, of both litigation and other compensation processes, reported that participants expressed frustration with how many complex forms they had to complete and the volume of paperwork which needed to be submitted to support their claims [36, 37, 46, 68, 106, 109–112].

Participants also reported difficulties with answering certain questions required to apply for disaster compensation from local governments and other agencies [68] or found it difficult to complete the necessary paperwork and documentation when they were living in temporary accommodation with limited access to computers or telephones [46]. Others struggled to provide the necessary documentation required [46, 107–111, 113, 114]. For example, many lacked documentation of their work status and income due to ambiguous or illegal employment relationships [113, 114] or were physically unable to submit necessary documents due to having lost them to disasters such as fires or floods [46]. Documents related to proof of home ownership could also be difficult to procure (e.g. when living in homes belonging to family members) [115–117] leading some to be ineligible for compensation.

The length of compensation processes was also a key challenge area for participants. Although one quantitative study [88] found that the perception that litigation had taken too long was not associated with depression, PTSD or anxiety, participants across the majority of studies were critical of the length of compensation processes [36, 37, 68, 74, 92, 104, 106, 108, 109, 115, 117–125]. Some participants felt that the process took such a long time that even if they were to receive compensation, it would be too late to help [123]. Waiting for cash settlements forced some to stay in damaged, mouldy, cramped, or otherwise uninhabitable homes [46, 117, 119] and for others whose livelihoods had been affected, the wait for compensation meant periods with no income [36, 125]. The claims process placed substantial demands on financial resources [46, 114] and the long wait for compensation prevented some from being able to purchase a home [92] or come to terms with the grief of having lost loved ones [109].

Several other challenges of the compensation process were only described in one or two studies each; for this reason, it was deemed that there were insufficient data for these challenges to have individual themes, but they are still worthy of discussion and so are presented together here under the theme ‘other challenges’. These included language barriers in communicating with those involved in the claims process [37, 113]; being taxed on damages awarded [86]; feeling rushed into making decisions about the future [105, 127]; fear of deportation if they approached assistance centres [37]; feeling discouraged from applying for compensation after hearing about others’ rejections [115]; and grief making it too difficult to assemble the information required to file claims [37].

In summary, the weight of evidence included thus far indicates that many experiences of compensation processes are negative, involving stress across multiple parts of the process, and informing a broad range of negative mental health and wellbeing impacts at the individual level. However, we note that it is possible that those with negative experiences and therefore strong feelings about the issue may have been more likely to participate in the research; additionally, the methods of data collection did not always allow for the identification of positive experiences. This is discussed further in the Limitations section of this review. The data also sheds light on system-wide challenges experienced across compensation processes.

### Procedural (in)justices

The next overarching theme from our analysis explores the mental health and wellbeing impacts of real or perceived compensation-related procedural, interpersonal, and distributive (in)justices.

First, procedural (in)justices have the potential to be perceived or experienced as positive by some individuals and communities, whilst being perceived and experienced as negative by other individuals or communities simultaneously. This review demonstrated that compensation system-related procedural injustices frequently impact individual health and wellbeing and community relations in negative ways. It identified five types of procedural justice (informational; lack of eligibility; lack of interagency cooperation; lack of understanding communities; and insufficient payment), all of which will be described in greater detail below.

For example, many studies focusing on compensation funds and disaster assistance reported challenges of informational (in)justices relating to poor communication and a lack of accurate, easily accessible information which left them confused about what was actually available to them; what compensation they were eligible to apply for; and later, the status of their claims [36, 37, 46, 68, 90, 111–115, 120, 124, 126, 127]. Others found that misinformation or conflicting information were provided [36, 46, 104, 105, 112, 116, 121]. Poor communication was reported across studies of all different compensation types. Lack of communication and updates throughout the process were seen to be re-traumatising by participants in one study rated as very high-quality [127] and left participants feeling forgotten [115, 124]. Mayer et al. [90], in their qualitative study, suggested that lack of reliable information and clear standardised guidelines caused more damage to communities than anything else.

Another procedural (in)justice relates to the eligibility criteria and assessment of claims within the compensation process. Participants in several studies shared their disappointment and anger at the multiple exclusionary criteria which left some ineligible for compensation, leading them to perceive the compensation process as non-inclusive [37, 92, 112, 113, 115, 116, 120, 121, 124, 128, 129]; this often resulted in a perceived increase in wealth disparities. For example, eligibility criteria were perceived to disproportionately disadvantage immigrant and undocumented workers [37, 113], Black people [115], homeowners with uninsured or under-insured properties [121] and live-in tenants [124]. Claim assessments contributed to long delays in receiving compensation [46] and there was a reported lack of transparency in the assessment process [112]. Additional problems included assessments by insurance companies and assessors not matching [110]; damage assessment negotiations [105]; having to get reassessments which were slow and involved explaining the situation each time [121]; disagreements over the valuation of homes [119]; and disputes over measurements of land and housing damage [121]. Additionally, assessors who went out to properties were often stressed themselves and not experienced in dealing with traumatised people, according to one study rated as very high-quality [127]. Participants involved in the distribution of compensation echoed thoughts about confusion and lack of transparency [117, 125].

Additionally, lack of inter-agency cooperation was identified as a procedural justice issue. For compensation processes relating to disaster assistance, there were often a number of different agencies involved in the process, which caused tensions and confusion and often made the process more complicated if there was little cooperation between them [37, 120, 128, 130]. Poor cooperation between local and outside organisations was viewed as a source of disruption not only to local social norms but also to ongoing response efforts in one qualitative study [130]. In some cases, poor coordination led to benefits being duplicated which fuelled perceived distributional inequities [37, 120].

Participants in several studies of disaster assistance also reported that those in charge of compensation payments lacked fundamental understanding of their specific communities and local needs, which made the process appear unfair [36, 112, 120, 130–132]. In particular, participants in rural and coastal communities appeared to believe that individuals from large cities who were involved in their compensation claims had inadequate understanding of their unique situation [36, 112]. For example, in an American study of a wildfire [112], participants felt that officials in charge of their disaster pay-outs – who were based in cities – had a poor understanding of the rural west and lacked capacity to understand the disaster needs, local values, and severity of impacts unique to this type of community. Similarly, participants who had been affected by the Deepwater Horizon oil spill [36] felt that the person in charge of pay-outs (who was from New York) did not understand what it was like to live in the Gulf of Mexico, the regional economy, or the importance of the beach and water to the community; as a result, the person in charge was perceived to be overwhelmed by the number and nature of the claims, making the compensation process appear chaotic. Additionally, participants in one Sri Lankan study of tsunami survivors [120] pointed out a lack of understanding of sociocultural, religious and gender norms in the provision of rehousing as compensation: in this study, displaced Muslim women had to live much more ‘open’ lives than they had been used to and risked becoming victims of gender-based violence.

Finally, participants in several studies of both litigation and disaster assistance described their pay-outs as inadequate [36, 68, 86, 108, 114, 122, 126–128, 133]. Participants in one study felt their pay-outs would be better used for disaster prevention in the community rather than divided into small sums for each resident [68], whilst participants in another study where individual assistance was withheld [112] believed individual sums could have helped them deal with the impacts of the disaster. In some cases [133] participants were forced to leave their jobs and communities due to the lack of sufficient compensation.

### Interpersonal (in)justice

Interpersonal (in)justices have the potential to increase inequities and perceived inequities in compensation processes. For example, respondents provided evidence of perceived corruption and politicisation of compensation processes. Several studies reported that the (non-litigation) compensation process was perceived to be corrupted and politicised [108, 118, 120, 122, 126, 129, 130, 133–137]. The process was described as subject to manipulation [120] and politically motivated, with aid perceived to be diverted towards voters and supporters of the political elites in charge [118, 126, 134–136]. Others believed that well-connected households received more support than those who needed it the most [108, 133, 137]. Participants in these studies described the perceived corruption of the compensation to be distressing and to hinder them in accessing resources, but no studies investigated whether there was a direct statistical association between mental health and perceived corruption/politicisation of disaster assistance.

Similarly, real or perceived interpersonal (in)justices undermine trust in compensation systems. Studies of both litigation and compensation funds/disaster assistance reported low levels of trust in officials. Being a litigant significantly predicted lack of trust in institutions [93], and perceived fairness of disaster assistance distribution was significantly associated with trust in political leadership and social trust [134, 135]. Perhaps unsurprisingly, then, the numerous problems and challenges associated with the compensation process lowered many participants’ trust in authorities and officials [68, 103, 108, 123, 125, 126, 131, 133, 138]. Trust in authorities and officials involved in the compensation distribution was often low due to the perception they were ‘working for’ the companies responsible for the disaster [36] and the perception that promises had been broken [138], as well as the lack of apologies [103]. Loss of trust in the legal system is highlighted in one study by a participant’s comment that their disappointment over not having their claim paid *“wasn’t as profound to me as the loss of confidence in our legal system”* [138, p.665].

### Distributive (in)justice

Prominent in the data was (perceived lack of) distributive justice, i.e. the fair distribution of resources, and its impact on communities. Many studies – of litigation, compensation funds and disaster assistance – reported conflicts, divisions and perceived injustice arising due to unequal compensation [36, 37, 90, 92, 109, 112, 114, 117–120, 125, 132, 133, 140–142]. For some, the compensation system was the most commonly identified source of frustration and barrier to recovery [141] and the source of most of the friction in communities [142].

Multiple reasons were offered for why compensation disparities existed. Compensation was perceived to vary depending on where people lived [114, 132], evacuation status [132], amount of damages [132], job status [132], home ownership status [96], type of job [114, 125], expected lifetime earnings [37], media coverage [112], number of claims made [90, 121] and gender, race and class [115].

Distributive (in)justice provides a useful lens through which to consider community-level impacts of compensation systems. Data from the review indicated a number of areas where distributive (in)justice impacts the health and wellbeing outcomes of communities. For example, several studies highlighted the perception that seemingly arbitrary decisions were at play. Many studies noted a perceived lack of transparency in the compensation distribution process [36, 90, 114, 120, 133]. Participants described a number of inconsistencies in compensation, even between people in very similar or identical situations, which made compensation distribution choices appear arbitrary [36, 90, 114, 131, 133, 141]. Those who received less reported feeling anger and stress stemming from the feeling they had been treated unjustly by authorities in one very high-quality qualitative study [131]. Several studies [36, 90, 141] found that employees often received compensation while those who employed them struggled through longer and more complex processes, causing tensions within the communities and the perception that business owners were unfairly discriminated against. The seeming randomness of compensation distribution was attributed to the whims of the individual claims processors by participants in another qualitative study [90].

Other studies identified a perceived lack of ‘deservingness’ and fraudulent claims. Several studies reported that participants perceived that many of those awarded compensation did not deserve it; they questioned the moral authority of those who received compensation and, in some cases, believed others had made fraudulent claims. Some raised questions of deservingness [37] while in other studies, community members were believed to have taken advantage of the system by filing for losses they did not quality for [36, 108, 114, 119, 122]; claiming compensation for damages they had not received [36]; claiming losses far in excess of their net worth [108]; leaving their villages for financial gain instead of staying to salvage the community [131]; and filing claims after doing short-term jobs they had never intended to return to [36]. Perceptions that others were taking advantage of the situation caused conflicts within communities [36]; in several studies of oil spills, participants reported resentment and hostility towards those who were perceived to be unfairly profiting from the disaster, nicknamed “spillionaires” [74, 90, 141]. However, one qualitative study noted that the frustration around supposedly accurate, deserving claims being denied outweighed frustrations over compensation funds being misappropriated [90].

When participants felt they were perceived by others as having taken advantage of the disaster and profited dishonestly, this caused them guilt about claiming compensation which made them feel alienated from their communities [102], maintained post-traumatic symptoms [102] and delayed their grief for lost loved ones [109].

Additionally, distributive (in)equalities fostered social comparisons with the potential to lead to envy and jealousy. The perceived arbitrariness of the compensation process generated social comparisons and competition that resulted in a ‘corrosive community’ and impeded the recovery process. Participants in a number of studies described feelings of envy and jealousy towards others who had received more than them [90, 114, 119, 120, 130, 132. 141]. As one participant stated, *“there was an awful lot of*, *“I hear so-and-so got this much from the claims process and I don’t understand why I couldn’t get at least that much”. That was the disastrous part of the claims process”* [90, p.378]. Some described it as stressful to see other people in the community spending large sums of money, while their own claims were delayed or denied [90]. Others described being the object of others’ envy because of the compensation they received [90, 92, 102, 130, 132] which was extremely isolating [90, 92] and distressing [102].

Perceived distributive (in)justices also impact community and family relationships. Several studies noted how communities were joined, united and looking out for each other until issues of distributive justice emerged and communities began to disintegrate [114, 120, 123, 129, 130, 132]. The corrosive community effect included fragmentation of social groups and relationships and reduced trust in social institutions, with conflicts exacerbated by perceived inequalities in the compensation process [74, 90, 141]. The perception that certain residents were unfairly compensated in comparison with others pitted community members against one another [36, 74, 92, 114, 125, 130, 132], creating disruptions in the community that were *“immeasurable and irreversible”* [132, p.83]. The arguments over compensation made people less willing to interact with other community members and led some to reduce how much they socialised, leading to further isolation during a time when social support is needed [90]. Good and very high-quality studies reported that divisions within communities could also arise due to disagreements around blame, responsibility and wanting justice from those responsible for the disaster [129, 131].

We found a small number of quantitative findings relating to community relationships. Perceived unfairness of the compensation process was significantly associated with viewing communities as fragmented in studies rated very high-quality and moderate quality respectively [98, 143] and lower trust in the benevolence of others and efficacy of mutual helping behaviour [98]. Pursuit of litigation was the strongest indicator of post-Hurricane Katrina relationships in one study [144] and litigation stress was the strongest predictor of perceptions of chronic community damage in another [93].

Relationships within families were also reported to suffer due to the compensation process in both quantitative and qualitative studies, with tensions around compensation causing arguments [144] and sometimes even leading to the dissolution of families [114, 121]. Altercations with extended families also occurred, sometimes due to arguments over money spent, demands for a share of the compensation, or requests for loans or gifts [108]. Others described uneasiness over having to demand relatives bill them for food and accommodation in order to claim their compensation [119].

We also found that a sudden influx of money had the potential to impact communities in a negative manner. In several studies, compensation had been awarded in large, one-off payments and the sudden availability of large sums of money was perceived to be disruptive to communities [36, 90, 116]. Especially among lower-income populations, many felt the sudden influx of cash into communities with previously limited resources was disruptive and created a disincentive to work, disrupting the local economy and creating community divisions [36]. Budgeting new money that arrived in a lump sum was difficult and misspending it could lead to accusations of fraud and threats of assistance being cut off [116].

Finally, other impacts of perceived distributive injustice included negative impacts on businesses, with differences in pay-outs creating growth opportunities for some businesses while constraining others [90]; those with less money to start with being less able to start recovery without help [117]; and perceived unfairness of house buyouts for some but not others making life more difficult for those who were not bought out [128, 131, 145]. For example, those left behind who were not eligible for the buyouts felt they were unsafe and exposed due to the tearing down of bought-out houses and perceived that their property values had been lowered [128]. We note that these findings emerge from participants who did not receive buyouts themselves, and would like to clarify that we do not claim that buyout schemes are not good for those who receive them; rather, they can negatively impact on those who are left behind.

### Positive findings

Although all studies described challenges, problems and stressors, a minority also reported some positive findings. However, as we have previously noted, the fact that a smaller number of positive findings than negative findings were reported does not necessarily indicate that compensation processes have primarily negative impacts. It is possible that those with strongly negative perceptions of the process might be more likely to volunteer to take part in compensation-related research.

Quantitative studies suggested that when compensation was received, with no unresolved issues and satisfaction with the process, mental health outcomes were improved [95–98]. Some participants reported being grateful for the formal assistance they did receive [109, 118, 121, 137]. Compensation received was believed to have helped participants restart their lives [118], recover from the disaster [121, 125], resume livelihood activities [125], keep their businesses open [36], build homes or purchase new properties in safer locations [121] and mitigate loss and damage [137]. Property buyouts were seen as a ‘way out’ for some [128].

Some participants in three studies perceived the compensation process to have been straightforward [36, 121, 127]; however, this was only reported by those with small claims [36], with no disputes [121] and who received pay-outs quickly [127]. The compensation fund available through the Gulf Coast Claims Facility, which introduced quick payment options of $5000 for individuals and $25,000 for businesses with minimal requirements for documentation provided that claimants waived future claims against responsible parties [36], was spoken of positively.

Some positive impacts on communities were described. For example, emergent community leaders helped compensation applicants to navigate the process [110, 115] and people still helped their relatives, neighbours and friends where possible [122]. It also appeared that reduced trust in authorities and officials sometimes resulted in strengthening community bonds as it forced people to rely on their social networks rather than institutions [126, 133, 135].

## Discussion

This review of 66 studies synthesised research examining the social and psychological consequences that can arise during post-disaster compensation processes. Understanding these consequences can inform interventions to support those facing mental health challenges during the compensation process and inform effective policy and practice regarding the compensation system, potentially leading to improved wellbeing and thus more effective rebuilding of individual lives and communities. Below, we discuss how the findings of this review have developed understanding of the effects of the compensation process, before suggesting practical ways in which this understanding can be used to improve compensation systems.

While we found mixed evidence of a (statistical) association between poor mental health outcomes and the compensation process, studies of all designs found that the claims process was typically described very negatively and perceived as stressful and sometimes ‘retraumatising’ by participants. Perceptions of distributive injustice were sometimes perceived to lead to jealousy, competition and divisions within previously united communities. Some studies reported positive findings, such as gratitude, perceived helpfulness of compensation and strengthened community bonds – suggesting social fragmentation is perhaps not inevitable. We note that the observation that only a minority of studies reported positive effects should be interpreted with caution: in quantitative studies in particular, positive effects were not always measured.

Overall, participants found both litigation and the process of applying for disaster assistance compensation to be stressful; the processes caused disappointment and anger and placed substantial demands on emotional resources. Some even described litigation as being equal to or more stressful than the disaster itself, supporting Hadler’s [146] description of *the tyranny of torts*, stating the demands placed on litigants to prove the magnitude of their symptoms are likely to reduce litigants’ ability to cope. Non-disaster-related literature has also reported mixed findings on the impact of the compensation process on mental ill-health [21, 147]. Many participants had difficulties navigating the compensation system, citing similar aspects of the process that individuals struggle with in non-disaster contexts [148]. Some studies we reviewed noted that the documentation needed to apply for compensation could disproportionately disadvantage certain groups – for example, immigrants, undocumented workers, and communities with particular sociocultural living arrangement norms with houses passed down through families without official documentation. This echoes previous research suggesting that systemic social inequities shape vulnerability, and people vulnerable before a disaster tend to suffer more after a disaster and experience worse mental health outcomes [149]. The issue of lack of paperwork and documentation is challenging to address: needing so much documentation creates a barrier for those who lack it, but if no documentation were required this would potentially encourage fraud [37]. Compensation regulators should consider how best to achieve a balance between the two. We suggest that sensitivity towards culture, immigrant status, gender and race needs to be promoted in the provision of disaster assistance to ensure that it can be accessed and utilised by specific potentially vulnerable groups [120]. In the aftermath of a disaster, researchers could be helpful in identifying particularly vulnerable groups who might struggle with documentation and establishing their specific needs.

Prominent in the data was the theme of distributive injustice and its impact on communities: studies reported conflicts between communities and other groups agencies/local governments; within communities; and within families. It is beyond the scope of this review to comment on whether compensation distribution genuinely was unfair or unjust in any of the contexts discussed, but what is important is that participants across studies *perceived* the process to be unfair, and also believed that feelings of jealousy and relative deprivation divided communities and hindered recovery. Participants across studies, in both litigation and disaster assistance studies, reported feuds, hostilities and damaged relationships. We found similar themes across the data – that is, studies on disaster assistance, compensation funds, and litigation all involved similar themes of long, confusing processes and perceived unfairness in compensation awards. Indeed, Mayer et al. [90] commented on how the Deepwater Horizon oil spill fund mirrored not only the outcomes of litigation (i.e., monetary award) but also the way in which litigation can disrupt communities. This finding supports our decision to include all types of compensation in this review.

Overall, our findings suggest that all nations should consider equitable processes for dealing with issues such as damage assessments, compensation pay-outs and litigation as part of their emergency preparedness and response plans [150, 151]. Preparation for this should include establishing in advance what kind of emergency assistance can be provided, and up to what limits; how eligibility criteria will be determined; how to meet the needs of those who are often left behind (e.g. undocumented workers); which agencies are responsible for managing compensation claims; how information will be disseminated (ensuring that it is communicated in a way that is accessible for everyone); whether there will be a flat rate or payments will be on the basis of actual costs incurred; how to identify those in need of assistance; and how to identify fraudulent claims [37, 150, 151]. These decisions can all be made at local levels *before* an incident occurs, pre-committing resources to be expended in the event of a disaster. However, we acknowledge that disasters are unpredictable by nature and that it is difficult to anticipate in advance what the most pressing needs might be [37]. For example, there could be unanticipated losses to infrastructure or particular population groups affected in unanticipated ways. We therefore suggest that, while it is important to make preparations in advance for how compensation might be managed, the unpredictable nature of disasters means that flexibility and ability to adapt to changing situations are also important.

Our findings suggest that psychosocial wellbeing of those affected by a disaster should be a key concern during the compensation process. Eriksen and de Vet [127] advocate for a trauma-informed approach to insurance claims handling, and we suggest a similar approach – one which is sensitive to the needs of vulnerable communities – for compensation claims would be useful. There are clear gaps in the literature relating to interventions; research on community reconciliation and resilience interventions would be useful. Disaster researchers could be extremely useful in improving the post-disaster compensation process. Sending researchers into the field to talk to disaster survivors and establish their needs would be extremely valuable. However, we also note that this can be stressful for the researchers themselves, particularly if they are involved in litigation research [152]. For that reason, we recommend that experienced researchers, or researchers with experienced supervisors – individuals better equipped to cope with the potential challenges from responsible parties – should be the ones conducting such research. We also note the potential for research fatigue in disaster-affected communities, who may be inundated for requests for research participation; this could be mitigated by using gatekeeper bodies to aid research coordination [153, 154]. If carried out sensitively, transparently and ethically, research with disaster-affected populations has the potential to improve the compensation process.

This is not to suggest that improving the post-disaster compensation process would eliminate post-disaster distress. There are a number of other stressors which many survivors face, such as continued media interest and perpetrators not being brought to justice [102] as well as changes to financial and living situations [29] and grieving for losses [109]. However, it does appear that reducing compensation stress might make post-disaster recovery considerably less distressing for disaster survivors. Improving the compensation process could also help to protect community resilience. When compensation processes cause divisions within communities, this undermines resilience by eroding trust, shared identity, and social capital essential for communities to recover from disasters [68]. However, engaging community members in the disaster assistance process to ensure compensation regulators understand unique local needs (e.g. needs specific to rurality or cultural norms), providing transparent and accessible information, and reducing inequities could strengthen communities’ capacities to withstand future events. Though it is undoubtedly difficult to achieve perfect equity, thoughtful compensation policies could strengthen, rather than corrode, the foundations of community resilience.

While the importance of understanding the impacts of compensation-seeking on community resilience have been well-reported [68] we also felt it was important to understand the impacts at an individual level and how negative mental health impacts could potentially be prevented. This is important for several reasons: firstly, in terms of the wellbeing of the individuals themselves. If compensation-seeking negatively affects mental health, this can substantially reduce quality of life and impair the ability to carry out daily tasks, as well as reduce self-esteem and damage interpersonal relationships [155]. The mental health of individuals can also have societal impacts [156], for example in terms of fractured community relationships, lost productivity at work and the economic costs of resources to meet the demand for mental healthcare. Below, we consider ways in which compensation processes could be improved to protect both individuals and communities.

### Implications

Based on the review’s findings, a number of policy recommendations emerge for managing post-disaster compensation processes in ways that may mitigate negative psychosocial impacts (see Additional Table 4: Recommendations based on the review’s findings).

First, relating to emergency planning, we suggest that local governments and policy-makers should make plans regarding the distribution of compensation prior to an extreme event occurring. For example, this might involve considering how to identify those most in need and how information will be disseminated.

Regarding communication, we suggest ensuring outreach to marginalised groups; ensuring communication is clear, consistent and transparent throughout the compensation process [157]; clearly outlining and justifying eligibility criteria for compensation; providing regular updates; and communicating with the media to ensure that they do not fuel resentment [158].

Relating to mental health, we suggest development of mental health services to help mitigate the potential negative effects of compensation-seeking on wellbeing and the offering of stepped care, with communities supported to look after themselves but also able to access formal mental health services if required.

Policies should be inclusive and consider the specific needs of affected communities. We suggest that consideration of how potentially vulnerable groups access compensation should be considered, with thought given to gender, race and socio-cultural norms. The values and norms of the specific communities affected by a disaster need to be taken into account; involving the affected communities in decision-making may be useful.

We suggest that fairness should be a key consideration throughout the compensation process. Using external agencies to monitor compensation distribution may be useful in ensuring the process is not corrupt or politicised. Processes for identifying and eliminating fraud would also be helpful. In litigation situations, mediation and arbitration by court-appointed experts may help ensure the process is fair.

Finally, in terms of directions for future research, we suggest that more research on interventions to enhance community resilience and reconcile divided communities is needed.

### Limitations

We report a number of limitations, both of our review itself and the studies included within. Firstly, it is notable that a number of our included studies were located through hand-searching (24/66, 36%) and not picked up by our database searches. This suggests that, although we searched multiple databases using a search strategy consisting of 83 unique search terms, it was insufficient to capture all relevant literature. Based on our knowledge of the literature from working in the disaster psychology field for many years, we suspect there are a number of papers looking broadly at post-disaster impacts on individual and community wellbeing, of which the compensation process is just one of many stressors discussed and therefore does not necessarily appear in titles, keywords or abstracts. A large review requiring a lot of time and resources would be needed to capture everything. However, our relatively consistent findings provide a strong evidence base of the potential negative psychosocial effects of post-disaster compensation processes. Secondly, the decision to limit the review to only English language studies limits the findings; future reviews might consider searching non-English literature. Third, we acknowledge the limitation of only one author screening the majority of citations. Due to 100% consensus between the first and fourth author on the 10% of citations which were double-screened, we did not carry out any more duplicate screening than this. However, we acknowledge that 10% is a small number to have double-screened and it is possible that due to human error citations may have been inaccurately excluded. Ideally, with adequate time and resources, 100% of citations would have been double-screened.

There are also several limitations of the literature. First, we found a lack of longitudinal research. It is therefore important that further research is carried out before we can fully understand the long-term impacts of compensation-seeking. Additionally, while we found much evidence that participants perceived the compensation process as unfair and unjust, few studies used statistical analysis to assess whether perceptions of compensation were associated with wellbeing. Many studies did not report the time and duration of exposure to compensation-related factors or did not clarify whether the process was ongoing at the time of data collection. Similar limitations have been noted in literature on compensation following injuries [159]. Additionally, similar to non-disaster compensation studies, many did not measure exposure to compensation procedures in an accurate way, instead simply asking participants if they were involved in compensation or litigation or had received assistance [24]. Involvement in the compensation process was often only asked at one time-point, not taking into account participants who may have dropped their claims – again, similar to findings in the non-disaster compensation literature [24]. Finally, we highlight the potential lack of representativeness within the study samples, and in particular how this might affect interpretation of how ‘positive’ and ‘negative’ findings are weighted within the literature. We noted that a minority of studies reported positive impacts of the compensation process. However, it is possible that those with strong negative feelings about the process would have been more likely to choose to participate in research, thus amplifying the more negative impacts of compensation-seeking. We also note that positive impacts were not always measured within the included studies. While most qualitative studies appeared to include at least some broad questions which would have allowed participants to speak about both positive and negative experiences, many of the measures used in quantitative studies would not have picked up on more positive impacts or allowed for identification of the potential nuances and complexities surrounding feelings towards the compensation process.

## Conclusion

Compensation is crucial in the rebuilding of lives and communities after disasters. However, the compensation process can unintentionally create social divisions and cause additional stress if not handled sensitively. Lengthy and complex bureaucratic procedures often result in inequitable distribution of compensation, leading to a sense of injustice and unfairness among survivors. This can exacerbate the trauma experienced during the disaster and weaken community resilience. Many survivors view the process as inadequate, inconsistent and biased, resulting in feelings of resentment, betrayal and a lack of faith in governments and other institutions. Delayed or inadequate compensation can create financial strain, making it difficult for survivors to rebuild their lives. Unequal distribution of compensation can lead to social and community tensions, resulting in conflicts, divisions, and a breakdown in social cohesion and community resilience. To minimise these negative impacts, it is crucial to ensure that compensation processes are efficient, transparent, and accessible to all survivors. Providing mental health guidance, support and resources throughout the process could help mitigate the psychological effects. By implementing thoughtful policies and efficient administration, post-disaster compensation can effectively repair material losses without further deteriorating the social fabric of communities (that is, the various social and cultural values, norms and relationships that shape communities). However, more research is required to explore interventions for fostering community reconciliation when compensation inequities occur. With efforts to address these issues, post-disaster compensation can serve its intended purpose of rebuilding lives and communities without inadvertently exacerbating mental health impacts and community divisions.

## Electronic supplementary material

Below is the link to the electronic supplementary material.

Supplementary Material 1: Additional Table 1. Caption:Characteristics of included studies

Supplementary Material 2: Additional Table 2. Caption:Compensation process details of included studies

Supplementary Material 3: Additional Table 3. Caption:A typology of compensation impacts

Supplementary Material 4: Additional Table 4. Caption:Recommendations based on the review’s findings

Supplementary Material 5: Appendix 1. This is the PRISMA checklist

Supplementary Material 6: Appendix 2. This is the search strategy

## Data Availability

Data is provided within the manuscript or supplementary information files.
