# Supplementary material for: Psychosocial impacts of post-disaster compensation processes: narrative systematic review
Source: BMC Psychol. 2024 Oct 7;12:539. doi: 10.1186/s40359-024-02025-9 (PMC11460075; doi:10.1186/s40359-024-02025-9)
Supplement: Supplementary file 1 — Supplementary Material 1: Additional Table 1. Caption:Characteristics of included studies [file 40359_2024_2025_MOESM1_ESM.docx]

**Additional Table 1. Characteristics of included studies**

| **Authors (year)** | **Article type** | **Country** | **Disaster setting and compensation type** | ***‘n’*** | **Key demographic characteristics** | **Design** | **Outcomes assessed** | **Funder** | **Quality** |
| --- | --- | --- | --- | --- | --- | --- | --- | --- | --- |
| Akbar & Aldrich (2016) [134] | Journal article;  Mixed-methods | Pakistan | 2010 Pakistan floods  Disaster assistance | 450 | Heads of affected households who received institutional aid in monetary form  Age not reported  88.9% male, 11.1% female | Mixed-methods: Cross-sectional survey, in-depth interviews | Flood damage; perceived leadership effectiveness; socio-demographics; satisfaction with fairness of disaster assistance distribution | Not reported | Moderate |
| Akbar & Aldrich (2017) [135] | Journal article;  Quantitative | Pakistan | 2010 Pakistan floods  Disaster assistance | 450 | Residents of flooded areas  Age not reported  88.9% male, 11.1% female | Cross-sectional survey | Education level, occupation, home stability, perceived fairness of disaster assistance, trauma experienced (personal injury, death of significant other, destruction), house damage, damage to other property, loss of vehicles, loss of documents, loss of livestock or crops, destruction of fish or poultry farm | Not reported | Good |
| Akbar et al. (2020)  [143] | Journal article;  Quantitative | Pakistan | 2010 Pakistan floods  Disaster assistance | 450 | Heads of affected households who received institutional aid in monetary form  Age, gender not reported | Cross-sectional survey | Socio-demographics; perceived fairness in distribution of disaster aid; flood experience; post-flood community cohesion | Not reported | Moderate |
| Akbar et al. (2023) [118] | Journal article;  Qualitative | Pakistan | 2010 Pakistan floods  Litigation | 20 | Farmers whose houses were damaged by the floods  Mean age 54.6, range 43-67  100% male | Interviews and focus groups | Life recovery experiences; contributors and inhibitors to life recovery | Not reported | Good |
| Arai (2012) [136] | Journal article;  Qualitative | Pakistan | 2010 Pakistan floods  Disaster assistance | ~500 | Residents of three flood-affected areas  Age and gender not reported; “male and female, from different age groups”, p.53 | Focus groups, supplemented by on-site observations of relief activities and in-depth interviews with a dozen development experts | Short-term and long-term socioeconomic and psychological impacts of the floods; challenges and needs identified in efforts to rebuild communities | Not reported | Moderate |
| Arata et al. (2000) [88] | Journal article;  Quantitative | United States of America | Exxon Valdez oil spill  Litigation | 125 | Commercial fishers  Age not reported  86.4% male, 13.6% female | Cross-sectional survey | Demographic data; anxiety, depression and PTSD assessed using SCL90-R; Modified Coping Strategies Scale; resource loss; job status; community cohesion; changes in physical health; involvement in litigation; perception that litigation process had taken too long; loss of money | Prince William Sound Regional Citizen’s Advisory Council | Very high |
| Barnes et al. (2002) [131] | Journal article;  Qualitative | United Kingdom | Chemical contamination incident  Disaster assistance | 23 | Age range 17-70  52% male,  48% female | Qualitative interviews | Interviews addressed knowledge about the quarry prior to January 2000; initial reaction to learning about contamination, concerns about health effects, views of public agency management of the incident, views on remaining in the village, and how to sustain the village in future | Not reported | Very high |
| Bartels et al. (2022) [107] | Journal article;  Qualitative | Haiti | Cholera outbreak  Disaster assistance | 77 | Individuals affected by the outbreak  Age group most commonly represented was 25-34 (29.9%)  64.9% male, 35.1% female | Open-ended story prompts | Story prompts allowed participants to share narratives on a variety of topics and those focusing on cholera were selected | Arts and Humanities Research Council; Social Sciences and Humanities Research Council | Good |
| Binder & Baker (2017) [130] | Journal article;  Qualitative | American Sāmoa | Earthquake and tsunami  Disaster assistance | Interviews: 22  Focus groups: 23 | Residents of the community affected by the disaster  Age not reported  Interviews: 36% male, 64% female  Focus groups: 22% male, 78% female | Interviews, focus groups and observational data, together comprising a descriptive case study | Experiences of the tsunami and recovery process | Not reported | Good |
| Binder et al. (2020) [2020] | Journal article;  Qualitative | United States of America | Hurricane Sandy  Disaster assistance | 37 | Homeowners in a coastal community in which many homes were bought out  Mean age 58.7 (range 27-82)  30% male, 70% female | Qualitative interviews and field notes kept by research team | Experiences of households on the periphery of home buyout zones; how the buyout has affected the community and individuals | National Science Foundation | Good |
| Bourova et al. (2022) [46] | Journal article;  Qualitative | Australia | Multiple – storm or hailstorm, bushfire, flood, lightning strike  Insurance claims | 30 | Individuals who had made claims relating to a disaster or extreme weather event  Age not reported  20% male, 80% female | Qualitative interviews | Experiences of making insurance claims in the aftermath of a disaster; adequacy of cash settlements or repairs provided; impacts of delays or other problems with the claims process | Australian Government through the Australian Research Council’s Discovery Projects funding scheme | Very high |
| Bowler et al. (1994) [89] | Journal article;  Quantitative | United States of America | Chemical disaster caused by a train derailment  Litigation | 90 exposed residents, 90 controls | People who lived near the spill vs. matched controls from a similar community  Mean age 44.6 (residents), 44.4 (controls)  61.1% male, 38.9% female | Cross-sectional | Structured clinical interview; medical and psychological history and symptoms; Impact of Event Scale; Mood Scale; Environmental Worry Scale; Perceived Social Support Scale; Perceived Control Scale; Profile of Mood States-Revised; blood pressure; pulse; saliva samples | Not reported | Good |
| Carroll et al. (2010) [104] | Journal article;  Qualitative | United Kingdom | 2005 floods in Carlisle  Insurance claims | 40 | Individuals whose homes had flooded and agency workers who helped them  Age range 30-70  35% male, 65% female | Focus groups and interviews | Perceptions and behaviours of respondents before, during and after the floods | St. Martins College provided financial support | Good |
| Cottar et al. (2021) [128] | Journal article;  Qualitative | Canada | Major flooding  Disaster assistance | 20 | Individuals who were knowledgeable about property buyouts and disaster recovery assistance programmes (e.g. government employees, insurance or real estate employees, disaster relief agencies, special interest groups – not homeowners  Age, gender not reported | Qualitative interviews | Awareness of buyouts or disaster recovery buyout programmes, factors influencing homeowner decisions to consider buyouts or rebuild, role of governments in policy implementation, criteria to evaluate programme effectiveness | Institute for Catastrophic Loss Reduction | Moderate |
| Croüail et al. (2020) [132] | Journal article;  Qualitative | Japan | Fukushima nuclear accident  Disaster assistance | 42 | Stakeholders including citizens involved in radiological protection, public health, agriculture and industry recovery, national authorities, companies involved in rehabilitation and decontamination, mayors, academics, local NGOs and residents  Age, gender not reported | Qualitative interviews | Health and environmental monitoring, radiological protection issues, pursuit of countermeasures in decontamination and waste management, meaning and understanding of evacuation criteria, living conditions of inhabitants | Not reported | Moderate |
| De la Hoz et al. (2008) [113] | Journal article;  Quantitative | United States of America | World Trade Center terrorist attacks  Disaster assistance | 168 | Immigrant rescue and recovery workers, including US-born (n=93), Polish (n=12), Latinos (n=63)  Mean age 44.6 (US-born), 53.3 (Polish), 42.9 (Latinos)  % female: 15.7% (US-born), 20.5% (Polish), 41.2% (Latinos) | Clinical evaluation | Demographic, occupational, socioeconomic and clinical characteristics evaluated using ‘item lists’ | WTC Medical Monitoring and Treatment Program funded by the National Institute for Occupational Safety and Health | Poor |
| Dixon & Kaganoff Stern (2004) [37] | Monograph;  Qualitative | United States of America | World Trade Center terrorist attacks  All compensation types | Not reported | Senior individuals in organisations affected by the attacks  Age, gender not reported | Interviews | Perspectives on losses suffered and compensation available; views on what kinds of compensation policies should be put in place for the future | RAND Institute for Civil Justice | Poor |
| Edgeley & Paveglio (2017) [112] | Journal article;  Qualitative | United States of America | Carlton Complex wildfire in Washington  Disaster assistance | 87 | Professionals and local residents  Age, gender not reported | Interviews | Experiences with the fire; perspectives about fire management or suppression; wildfire impacts to the respondent or broader population; recovery efforts | US Department of Agriculture – National Institute of Food and Agriculture; US Forest Service, Rocky Mountain Research Station | Moderate |
| Eriksen & de Vet (2021) [127] | Journal article;  Qualitative | Australia | Bushfires  Litigation or insurance claims | 17 | Bushfire survivors  Age range 30-80  Gender not reported | Qualitative interviews | Lived experiences of bushfires, insurance and longer-term recovery | Australian Research Council | Very high |
| Eyles et al. (1993) [119] | Journal article;  Qualitative | Canada | Hagersville tire fire  Disaster assistance | 43 | Residents of Hagersville  Age, gender not reported | Qualitative interviews | Experiences of the fire, experiences of the clean-up operation, how people coped with the event, impact of the fire | Environment Ontario | Good |
| FitzGerald et al. (2019) [140] | Journal article;  Quantitative | Australia | Queensland floods  Disaster assistance | 327 | Residents of areas affected by the floods  Mean age 57  49% male, 51% female | Cross-sectional survey | Social, economic, demographic, physical health and mental health information; movement within flooded or non-flooded areas; ongoing effects on physical or mental health; GHQ-12 to assess perceived health status; open-ended question asking about general flood experiences | Trauma and Disaster Management Research Grant Scheme funded by the National Critical Care and Trauma Response Centre Trust Fund | Good |
| Flagg (2017) [144] | Journal article;  Quantitative | United States of America | Hurricane Katrina  Litigation | 2,333 | Residents of Mississippi and Louisiana  Mean age 53.57, age 18-90  47% male | Cross-sectional survey | Damage to residence; residential changes; home insurance stress; flood insurance stress; chemical exposure; litigation; organisational involvement; family arguments; likelihood of neighbours helping each other out; impact of faith-based volunteer groups | Rockefeller Foundation; Bill and Melinda Gates Foundation coordinated through the Social Science Research Council | Moderate |
| Flocks & Davies (2014) [36] | Journal article;  Qualitative | United States of America | Deepwater Horizon oil spill  Claims facilities implemented to alleviate trauma on affected communities caused by prolonged litigation | 93 interviews, 20 focus groups | Residents in Gulf Coast counties  Age, gender not reported | Interviews, focus groups | Focus groups: Nature of local resiliency to disasters; impact of the disaster on their communities  Interviews: how the impact of the disaster compared to previous natural disasters; what characteristics of a community make it resilient and most vulnerable to technological disasters; overall and current impact of the disaster on the community; how effective the response was at various levels of government; satisfaction with claims process; suggestions for improving claims process; barriers to improving the claims process | Deepwater Horizon Research Consortia from the National Institute of Environmental Health Sciences | Poor |
| Forster et al. (2022) [133] | Journal article;  Qualitative | Dominica | Hurricane Maria  Disaster assistance | 22 interviews; 9 took part in focus groups | Representatives of government departments; members of the community including fishers, fish vendors, coastal tourism workers; village council workers; Parish Fisheries Cooperative; youth group members  Age, gender not reported | Qualitative interviews and focus groups | Questions exploring  what is meant by recovery, how hurricane impacts different aspects of wellbeing (material, subjective, relational), relationships within the community and externally, future aspirations for individuals and the community | Global Challenges Research Fund awarded by the University of East Anglia | Good |
| Frerks & Klem (2005) [120] | Report;  Qualitative | Sri Lanka | Tsunami  Government compensation scheme | 57 | Key informants, tsunami survivors, volunteers, Army units  Age, gender not reported | Observational research and qualitative interviews | Not reported | Not reported | Poor |
| Gill et al. (2016) [100] | Journal article;  Mixed-methods | United States of America | Exxon Valdez oil spill  Litigation | Not reported | Residents of Cordova, Alaska  Age, gender not reported | Longitudinal mixed-methods approach incorporating document review, observations, interviews, surveys | Quantitative: Demographics; community attachment; social disruption; psychological stress (Impact of Event Scale); depression; self-efficacy; resource loss; attitudes toward litigation  Qualitative: Community attachment; oil spill experience; resource loss; community disruption; lifestyle changes; litigation | National Science Foundation, Arctic Social Science Division; Natural Hazards Center, Institute of Behavioral Science, University of Colorado; Coastal Resource and Resiliency Center and Department of Sociology and Anthropology, University of South Alabama; Department of Sociology, Center for the Study of Disasters and Extreme Events, and College of Arts & Sciences – Oklahoma Sate University | Moderate |
| Green et al. (1990) [23] | Journal article;  Quantitative | United States of America | Buffalo Creek dam collapse  Litigation | 121 litigants, 78 non-litigants, 50 non-exposed  Mean age: 53.2 (litigants), 52.6 (non-litigants), 55.6 (non-exposed)  Litigants: 39% male, 61% female  Non-litigants: 31% male, 69% female  Non-exposed: 48% male, 52% female | Adults involved in lawsuit against the company; adults exposed to the disaster but not involved in the original lawsuit; convenience sample of people not exposed | Cross-sectional structured clinical interviews | Structured Clinical Interview for DSM-III to assess PTSD; Psychiatric Evaluation Form to assess symptoms such as anxiety and depression; Symptom-Checklist-90R to assess extent of symptoms in the past week; Progress Evaluation Scales to assess functioning | National Institute of Mental Health | Good |
| Hadfield (2008) [139] | Journal article;  Mixed-methods | United States of America | World Trade Center terrorist attacks  Victim Compensation Fund or litigation | 34 interviews, 155 surveys | Surveys: People who had lost a family member or were injured in the attacks  Interviews: 30 of the survey participants, 4 people publicly involved in efforts to reform the Victim Compensation Fund or shape public response to the disaster  Age, gender not reported | Mixed-methods: Qualitative interviews and cross-sectional survey | Experiences with the Victim Compensation Fund and the legal system; perceptions of who shared responsibility for losses suffered; reasons behind decision to accept compensation from the Victim Compensation Fund or decision to pursue litigation instead | Center for Advanced Study; the Mellon Foundation; USC Law School | Moderate |
| He et al. (2021) [121] | Journal article;  Mixed-methods | New Zealand | Canterbury earthquakes  Disaster assistance | 32 | Relocated households  Age, gender not reported | Mixed-methods: Interview and questionnaire | Post-earthquake experiences of relocation and recovery; attitude towards government policies | Higher Degree Research Funding Program at the School of Earth and Environmental Sciences at the University of Queensland | Moderate |
| Heyse (2001) [103] | Doctoral thesis;  Quantitative | United States of America | Buffalo Creek flood  Not reported | 103 (although only data from 100 was complete and therefore included) | Residents of Fort Collins affected by the flood  43% male, 57% female  Median age group was in the range of 35-44 | Cross-sectional survey | Event impact (financial and relative damage, life disruption); justice (compensation received, process fairness, interpersonal fairness, quality of explanations; stress levels for the first year after the flood; recent stress; evaluations of the City; satisfaction with compensation in the form of relief and mitigation | Not reported | Good |
| Jones et al. (2018) [129] | Journal article;  Mixed-methods | Mexico | 2009 Hermosillo ABC Day Care Center fire  2014 Cananea copper mine spill  Government compensation schemes | Fire: 226 parents/  caretakers (unclear how many others)  Oil spill: 114 farmers and other residents (unclear how many officials) | Fire: Parents and caretakers from families affected by the fire; other semi-structured interviews with parents, caretakers and citizens  Oil spill: Farmers, residents, official and key informants  Age, gender not reported | Ethnography: Combination of ethnographic, survey, governmental and newspaper data | Fire: interviews to understand how grief varied among different categories of people associated with the event; how people differed in their response to complicated grief; the event itself; reactions to the event; efforts to organise and search for justice  Oil spill: Interviews to understand people’s perceptions of the problems and how they have interacted with others in their recovery  Both studies asked about the impact of the event on individual and household daily activity; economic wellbeing; social networks; physical health and mental health | Natural Hazards Center at the University of Colorado; CONACYT through el Instituto de Geología/UNAM; US National Institutes for Health; National Science Foundation | Good |
| Joseph & Jaswal (2014) [108] | Journal article;  Mixed-methods | India | Mumbai terror attack  Disaster assistance | 231 family profiles, 94 individual surveys  Interviews: 40 | Terror attack survivors  Age, gender not reported | Mixed-methods: questionnaire, qualitative case studies constructed from process records and interviews | World Health Organization’s SRQ20  Case studies of those reporting significant distress were reviewed to understand factors contributing to distress  Case studies followed up with interviews to seek understanding of the situation | Not reported | Moderate |
| Kammer-bauer & Wamsler (2017) [110] | Journal article;  Mixed-methods | Germany | Floods  Disaster assistance | 8 interviews  53 surveys | Key informants from regional and local institutions, surveys, non-profit organisations and affected residents  Interviews: Age, gender not reported  Survey: 66% female, age not reported | Mixed-methods: Interviews, survey of households, reviews of documentation, walk-through analyses, observation, geographical analyses | Not reported | Swedish Research Council FORMAS | Moderate |
| Kaniasty (2012) [98] | Journal article;  Quantitative | Poland | Flood  Disaster assistance | 285 | Residents of the Polish city Opole and surrounding villages  Mean age 48 (range 18-87)  35% male, 65% female | Cross-sectional structured interviews | Disaster exposure; material losses; post-disaster altruism in the community; social support; post-disaster social bitterness (dissatisfaction with aid, social disaffection, interpersonal conflicts); community cohesion; withdrawal from interpersonal contacts; belief in benevolence of people; belief in efficacy of mutual helping | Polish Committee for Scientific Research; State System of Higher Education, Pennsylvania; and the International Research Exchanges Board | Very high |
| Komlósi et al. (2015) [105] | Journal article;  Mixed-methods | Hungary | ‘Red sludge disaster’ (industrial catastrophe)  Disaster assistance | Not reported | Not reported | Mixed-methods: analysis of published documents of events, reports and notes of volunteer psychologists, and results of two studies | Not reported | Not reported | Moderate |
| Marshall et al. (2004) [99] | Journal article;  Mixed-methods | United States of America | Exxon Valdez oil spill  Litigation | Not reported | Residents of Cordova, Alaska  Age, gender not reported | Longitudinal | Impact of Events Scale to assess PTSD and stress-related illness | National Science Foundation, Polar Social Science Division | Moderate |
| Mayer et al. (2015) [90] | Journal article;  Qualitative | United States of America | Deepwater Horizon oil spill  Compensation scheme or litigation | Focus groups: 20 groups consisting of 4-10 people  Interviews: 103 | Residents of four Gulf Coast communities  Age, gender not reported | Focus groups and interviews | Focus groups: Nature of local resiliency to disasters; overall impact of the spill on communities  Interviews: Experience of the spill, from initial reactions to news of the spill to efforts to emotionally and financially recover | National Institute of Environmental Health Sciences as part of the Deepwater Horizon Research Consortium | Good |
| Min et al. (2010) [97] | Conference abstract;  Quantitative | South Korea | Gangwon Province flood  Not reported | 57 | Residents of the village  Age not reported  44% male, 56% female | Follow-up study | SF-36-K to assess health-related quality of life; Beck Depression Index to assess depression; Psychosocial Well-being Index to assess wellbeing; MMPI-PTSD to assess PTSD; compensation satisfaction | Not reported | Moderate |
| Morris et al. (2013) [141] | Journal article;  Qualitative | United States of America | Deepwater Horizon oil spill  Not reported | 90 interviews, 19 focus groups | Residents of coastal communities dependent on tourism and seafood harvesting  Age, gender not reported | Interviews and focus groups | Social and economic impacts after the spill; challenges in the compensation process; potential strategies for recovery | National Institute of Environmental Health Sciences as part of the Deepwater Horizon Research Consortium | Moderate |
| Muzamil et al. (2021) [122] | Journal article;  Mixed-methods | Pakistan | 2010 flood in Swat  Disaster assistance | Survey: 108  Interviews: 12  Focus groups: 3 groups of 6-9 people | Survey: Flood-affected communities  Interviews: Government officials, politicians, NGO members, journalists, academics, community activists  Focus groups: Male farmers, shopkeepers, teachers, handymen, merchants and youth  Age, gender not reported | Mixed-methods: interviews, focus groups, survey | Survey: Flood impacts, impact of armed conflict with the Taliban, recovery initiatives, institutional responses  Interviews: Impacts of the flood, conflict, and deforestation  Focus groups: Institutional support, activities, challenges to the recovery process | Pakistani Government Scholarship and University Postgraduate Award at the University of Western Australia | Moderate |
| Ng (2016) [126] | Journal article;  Not reported | Thailand | Flood  Disaster assistance | Not reported | Residents of Ayutthaya  Age, gender not reported | Not reported | Not reported | NUS Graduate Research Support Scheme | Poor |
| Palinkas et al. (1993) [142] | Journal article;  Quantitative | United States of America | Exxon Valdez oil spill  Disaster assistance | 594 | Residents of 13 Alaskan communities (11 exposed to the oil spill and 2 control communities)  68.8% of the population aged 18-44  50.1% male | Cross-sectional survey | Socio-demographics; exposure to the oil spill; impact of the spill on social relations; impact of the spill on traditional subsistence production and distribution activities; CES-D to assess depression; modified version of the Diagnostic Interview Schedule to assess anxiety and PTSD; perceptions of change in substance use and domestic violence in the community; physical health status | ‘Oiled Mayors’ Sub-committee of the Alaska Conference of Mayors to Impact Assessment, Inc | Good |
| Patel (2022) [68] | Book chapter;  Qualitative | United Kingdom | 2013-2014 floods in Kent and Surrey  Disaster assistance | 77 | Residents of two flood-affected counties  Age group most represented was 55+ (62%)  38% male, 62% female | Focus groups and interviews | Number of years living in the community; number of floods experienced; flood experience; definitions of community resilience; aspects of the community that were helpful after the floods; elements of community resilience | King’s College London’s Graduate School’s International Award | Good |
| Picou (2009) [87] | Journal article;  Quantitative | United States of America | Exxon Valdez oil spill  Litigation | Not reported | Residents of Cordova, Alaska  Age, gender not reported | Mixed-methods: Longitudinal surveys and interviews | Impact of Event Scale to assess psychological stress; Center for Epidemiological Studies Depression Scale to assess depression | National Science Foundation; Office of Polar Programs; Prince William Sound Regional Citizens’ Advisory Council | Moderate |
| Picou & Gill (2000) [123] | Book chapter;  Mixed-methods | United States of America | Exxon Valdez oil spill  Litigation | Not reported | Residents of Alaskan communities  Age, gender not reported | Survey and ethnographic methods | Not reported | National Science Foundation; Earthwatch Center for Field Studies; Natural Hazards Resource and Applications Information Center; Prince William Sound Regional Citizens’ Advisory Council; partial support from College of Arts and Sciences, University of South Alabama and the Mississippi Agricultural and Forestry Experiment Station | Moderate |
| Picou & Hudson (2010) [91] | Journal article;  Quantitative | United States of America | Hurricane Katrina  Insurance claims and grants | 810 | Residents of two impacted counties  Mean age 55.1, range 18-90  52.5% female | Cross-sectional survey | CES-D scale to assess depression; Impact of Events Scale to assess psychological stress and PTSD symptoms; separation from family; residential damage; financial problems; claims filed to insurance companies | Rockefeller Foundation and the Bill and Melinda Gates Foundation funded through the Social Science Research Council | Moderate |
| Picou & Martin (2007) [87] | Report;  Quantitative | United States of America | Exxon Valdez oil spill  Litigation | 372 | Residents of Cordova, Alaska  Mean age approximately 50  54.8% male, 45.2% female | Longitudinal surveys | Impact of Events Scale; extrinsic resource loss distress; intrinsic resource loss; litigation disruption | National Science Foundation, Polar Social Science Division | Good |
| Picou et al. (2004) [93] | Journal article;  Quantitative | United States of America | Exxon Valdez oil spill  Litigation | 163 | Residents of Cordova, Alaska  Age, gender not reported | Cross-sectional survey | Work disruption; litigation stress; recreancy; oil spill risk; community attachment; community damage; intrusive stress | National Science Foundation, Polar Social Science Division; University of South Alabama; Mississippi Agricultural and Forestry Experiment Station | Moderate |
| Reid (2013) [124] | Journal article;  Qualitative | United States of America | Hurricane Katrina  Disaster assistance | 71 | Hurricane survivors  Mean age 43 (range 18-78)  61% female, 39% male | Qualitative interviews (multiple) and field observations | Evacuation experiences, use of social services programmes, housing situation | National Science Foundation | Good |
| Ritchie (2012) [106] | Journal article;  Qualitative | United States of America | Exxon Valdez oil spill  Litigation | 48 | Individuals affected by the disaster including businesspeople, government officials, fishermen, individuals prominently involved in litigation, mental health professionals  Mean age 51, range 28-85  46% male, 64% female | Qualitative interviews | Relationships between social capital and documented effects of technological disasters; personal and family background; commercial fishing experiences; subsistence lifestyles; ties to the environment | Not reported | Moderate |
| Ritchie & Long (2021) [94] | Journal article;  Quantitative | United States of America | Tennessee Valley Authority coal ash spill  Litigation | 716 | Residents of Roane County  44.1% aged 65+  56.9% female | Cross-sectional survey | Impact of Event avoidance behaviour subscale; personal relationship disruption; risk perceptions; perceptions of cleanup and restoration; adverse impacts of compensation process; event experience; community involvement; economic loss; socioeconomic status | National Science Foundation’s Division of Civil Mechanical and Manufacturing Innovation | Good |
| Ritchie et al. (2013) [138] | Journal article;  Qualitative | United States of America | Exxon Valdez oil spill  Litigation | Not reported | Residents of Cordova, Alaska  Age, gender not reported | Qualitative panel study | Not reported | National Science Foundation, Arctic Social Science Division; additional support from Natural Hazards Center, Institute of Behavioral Science, University of Colorado and the Department of Sociology and College of Arts & Sciences, Oklahoma State University | Moderate |
| Ritchie et al. (2018) [74] | Journal article;  Quantitative | United States of America | Deepwater Horizon oil spill  Litigation | 1,216 | Residents of coastal Alabama  Mean age 64 (post-weighted sample: median age 48)  Over 60% female (post-weighted sample: 50% female) | Cross-sectional survey | Involvement with claims, settlement, or litigation activity; experiences of compensation process; Impact of Events Scale to assess psychosocial stress | National Science Foundation’s Division of Polar Programs | Moderate |
| Sapat et al. (2023) [111] | Journal article;  Quantitative | United States of America | Concurrent disasters: Winter Storm Uri and COVID-19  Disaster assistance | 196 | Residents of five Texas counties  Age group most represented was 18-34 (49.5%)  49.5% male, 49.5% female, 1% other | Cross-sectional survey | Difficulty of applying for government assistance; reasons for difficulties; damage to residence; interactions with government officials; ability to cope; loss of power or water; previous disaster experience; socio-demographics | US National Science Foundation | Good |
| Scott et al. (1995) [102] | Journal article;  Mixed-methods | United Kingdom | Lockerbie bombing  Litigation | T1: 66  T2: 25 interviews, 10 surveys, 8 with limited data | Litigants assessed for medico-legal purposes  T2 sample: Mean age 46; 60% female | Mixed-methods: Survey, interviews, and authors discuss ‘other anecdotal reports’ but it is unclear where these are from | GHQ-28 to assess emotional state; Revised Impact of Event scale to assess intrusion and avoidance; life events; coping | Not reported | Good |
| Simington (2023) [115] | Journal article;  Qualitative | United States of America | Floods; Hurricanes Matthew and Florence  Disaster assistance | 37 | Residents of Marion County (including 15 non-Hispanic Black low-income heads of household and 22 community leaders)  Age not reported  Households: 13% male, 87% female  Community leaders: 32% male, 68% female | Qualitative interviews and ethnographic observations | Experience of living in the county; current financial situation and home life; current housing situation and neighbourhood; health; community perceptions about churches, politics, demographic change and crime; impact of the hurricanes; assistance received after hurricanes; challenges facing the community | Robert Wood Johnson Foundation | Moderate |
| Sodeyama et al. (2022) [95] | Journal article;  Quantitative | Japan | Fukushima nuclear disaster  Not reported but data suggests disaster assistance | 310 | Residents of Ikbaraki who had evacuated from Fukushima  Age, gender not reported | Cross-sectional survey | Socio-demographics; place of residence before the disaster; degree, cause and composition of damage; number of times evacuated; receiving or not receiving compensation; current health conditions; economic problems; work problems; neighbourhood problems; worries about hometown; depressive symptoms; PTSD symptoms; suicidal ideation | Not reported | Good |
| Sou et al. (2021) [117] | Journal article;  Qualitative | United States (Puerto Rico) | Hurricane Maria  Disaster assistance | 20 households (T1), 16 households (T2), 13 households (T3), 12 households (T4), 12 households (T5)  73 interviews | Residents of Ingenio, a neighbourhood in Puerto Rico  Households included men, women and children | Longitudinal qualitative methodology consisting of field visits | Field observations and interviews focusing on impact of hurricane and recovery strategies | Not reported | Good |
| Sterett (2012) [116] | Journal article;  Qualitative | United States of America | Hurricane Katrina  Disaster assistance | 90 in first wave, 82 in second wave | Displaced adults  Modal age range 40-50 for African American interviewees and 20-30 for White interviewees  44% male, 56% female | Qualitative interviews | Life before the hurricane; evacuation; how people came to Denver; how people accessed government resources | National Science Foundation | Good |
| Sungur & Kaya (2001) [101] | Journal article;  Quantitative | Turkey | Sivas massacre  Litigation | 79 | Individuals exposed to the disaster (27 people saved from the burning hotel, 34 people besieged at the Cultural Center, 18 health professionals who treated the injured  Age, gender not reported | Longitudinal surveys | Socio-demographics and personal/family history of trauma; Impact of Event Scale; DSM-III-R criteria used to assess PTSD; Beck Depression Inventory used to assess depression; Hamilton Depression Rating Scale used to assess severity of depression | Not reported | Moderate |
| Talbot (2011) [109] | PhD thesis;  Qualitative | United States of America | World Trade Center terrorist attacks  Disaster assistance | 12 | Adults who lost loved ones in the attacks  Mean age 45.5, range 28-59  25% male, 75% female | Qualitative interviews | Experiences of loss and grief; family and support system; coping; perceptions of media coverage | Fahs-Beck Fund for Research and Experimentation of New York Community Trust | Very high |
| Tsujiuchi (2021) [92] | Journal article;  Quantitative | Japan | Fukushima nuclear disaster  ‘Reparations’ | 2,862 | Families living in and evacuated from Fukushima prefecture  Age group most represented among compulsory evacuees and tsunami evacuees was 60-69 (29.4% of compulsory evacuees, 45% of tsunami evacuees)  Age group most represented by voluntary evacuees was 40-49 (30.9%)  Compulsory evacuees: 63.7% male, 34.7% female  Voluntary evacuees: 37.6% male, 60.1% female  Tsunami evacuees: 55.7% male, 43.6% female | Survey (cross-sectional analysis as well as comparisons to surveys from the past four years) | Impact of Event Scale-Revised to assess PTSD symptoms; experiences of the disaster; social consequences of evacuation; willingness to disclose evacuee status; satisfaction with family relationships; living condition; economic situation | Japan Society for the Promotion of Science KAKENHI | Moderate |
| Tsujiuchi et al. (2016) [96] | Journal article;  Quantitative | Japan | Fukushima nuclear disaster  Not reported | 350 | Households displaced from Fukushima  Mean age 54.2  47% male, 53% female | Cross-sectional survey | Socio-demographics; evacuation area; housing damage; health status (physical and mental health); Impact of Event Scale-Revised to assess symptoms of trauma; socio-economic disaster-related consequences (worries for livelihood, job loss, family split-up due to jobs/education needs/space restriction, loss of social ties, loss of social support, concerns for compensation); written narratives so participants could express in their own words the impact of the disaster | Disaster Relief Volunteer & Nonprofit Organization Support Fund 2012 by Central Community Chest of Japan; Japan Society for the Promotion of Science KAKENHI; Ministry of Education, Culture, Sports, Science & Technology Japan KIBANKEISEI 2021; Saitama Sogo Law Firm | Good |
| Van der Geest & Schindler (2016) [137] | Journal article;  Mixed-methods | Nepal | Landslide  Disaster assistance | 234 | Households in the affected area  Age not reported  Households headed predominantly by males (81.5%) although it is unclear if only the heads of household participated | Cross-sectional survey  Findings validated by expert interviews, focus groups and secondary sources | Livelihood activities; income; assets; food security; landslide loss and damage; effectiveness and costs of preventive and coping measures adopted; perceptions of vulnerability; recommendations for future actions that could be taken to protect people against landslide impacts | Asia-Pacific Network for Global Change Research | Moderate |
| Van Truong et al. (2021) [125] | Journal article;  Mixed-methods | Vietnam | Toxic chemical leak  ‘Remedial compensation’ | Survey: 250  Interviews: 40 | Households affected by the leak  Survey: Age not reported; 85% male, 15% female  Interviews: Age not reported; 75% male, 25% female | Cross-sectional survey and interviews | Survey: length of time out of work, impacts of the leak on household livelihoods, recovery strategies, government compensation  Interviews: How households responded to and recovered from the toxic leak | Vietnam National Foundation for Science and Technology | Moderate |
| Won et al. (2019) [114] | Journal article;  Qualitative | South Korea | Hebei Spirit oil spill  Compensation fund | 12 | Residents of the affected area  Mean age not reported, but all were aged 50+ except for one participant in their 40s  58% female | Qualitative interviews | Not reported | Ministry of Education of the Republic of Korea and the National Research Foundation of Korea | Moderate |
