# Supplementary material for: Psychosocial impacts of post-disaster compensation processes: narrative systematic review
Source: BMC Psychol. 2024 Oct 7;12:539. doi: 10.1186/s40359-024-02025-9 (PMC11460075; doi:10.1186/s40359-024-02025-9)
Supplement: Supplementary file 2 — Supplementary Material 2: Additional Table 2. Caption:Compensation process details of included studies [file 40359_2024_2025_MOESM2_ESM.docx]

**Additional Table 2. Compensation process details of included studies**

| **Authors (year)** | **Compensation type** | **Details of compensation process** | **Time period of data collection** | **Compensation status at the time of data collection** |
| --- | --- | --- | --- | --- |
| Akbar & Aldrich (2016) [134] | Assistance | The Government of Pakistan distributed Watan cards which could be used to withdraw up to 20,000 Pakistani rupees from any bank branch in Pakistan. | Floods began in July 2010; date of data collection not reported | Not reported |
| Akbar & Aldrich (2017) [135] | Assistance | Same as above (Akbar & Aldrich, 2017). Distribution of aid was carried out through local political elites. | Floods occurred in July-August 2010; data collected February-May 2013 | Not reported |
| Akbar et al. (2020) | Assistance | All individuals had received institutional aid in monetary form from government-led relief and rehabilitation agencies. | Floods began in July 2010; data collected January 2013 | Completed |
| Akbar et al. (2023) [118] | Litigation | 10 years post-oil spill, legal appeals to a $5 billion jury award to commercial fishers continued to be reviewed by the courts. | Floods occurred in July-August 2010; data collected August 2020 | Ongoing |
| Arai (2012) [136] | Assistance | By the end of October 2010 Pakistan had received approximately $1.7billion in donations from foreign countries, international organisations and private donors. The Pakistan government also initiated its own relief efforts. | Floods occurred in July-August 2010; data collected September 2010 | Not reported, assumed to be ongoing |
| Arata et al. (2000) [88] | Litigation | Legal appeals to a $5billion jury award to commercial fishers continued, at the time of publication, to be reviewed by the courts 10 years following the oil spill. | Oil spill occurred in March 1989; data collected in Fall 1995 | Ongoing |
| Barnes et al. (2002) [131] | Assistance | Imperial Chemical Industries (ICI), which owned the chemical plant responsible for chemical contamination, “quickly arranged” compensation packages (p. 2229) but the scheme was controversial because most of the village was divided into two compensation zones for compensation and house value protection. The ‘green zone’ was closest to the boundaries of the two quarries, and all residents here were offered £5000 each. The ‘blue zone’ consisted of most other houses in the village, where all residents were offered £2500 each. ICI offered to purchase the houses, at up to 20% above market value, of those in blue and green zones. A small number of houses within the village and many more outside the village but still close to the quarries received nothing. | Chemical levels identified as unsafe in December 1999; data collected June-July 2000 | Not reported |
| Bartels et al. (2022) [107] | Assistance | Seven months previously, the UN had outlined its plan to provide material assistance to individuals and families most affected by cholera. At the time of data collection, no participants or their families had received any compensation. | Outbreak began in October 2010; time of data collection not reported, but ‘approximately seven months after the UN outlined its two track New Approach, including the provision of material assistance’ | Ongoing |
| Binder & Baker (2017) [130] | Assistance | The post-disaster period reportedly involved outside aid interventions by national and international agencies and organisations. Housing assistance grants provided help for affected residents to rebuild their own homes and there was a pilot programme through which the agency oversaw construction of permanent homes; the latter was criticised due to the extremely high cost of homes built under the initiative and its failure to meet deadlines. | Incident occurred in September 2009; data collected January 2011 and March 2012 | Not reported |
| Binder et al. (2020) [2020] | Assistance | After Hurricane Sandy, the State of New York implemented a home buyout programme in three coastal communities. Between the hurricane and the time of data collection, over 300 homes in the buyout zone had been sold to the State and demolished. A small number of residents remained in the buyout zone either due to choosing not to participate or sales still being finalised. | Hurricane occurred in October-November 2012; data collected March-June 2016 | Ongoing |
| Bourova et al. (2022) [46] | Insurance claims | Participants were policy-holders who had made building or home contents insurance claims relating to loss or damage caused by a disaster or extreme weather event. | N/A – multiple events; data collected January-March 2021 | Some ongoing, some completed |
| Bowler et al. (1994) [89] | Litigation | Of the 220 oil spill-affected participants, 115 (52%) had individual legal representation related to the spill at the time of data collection, and 105 (48%) indicated no involvement in litigation. | Incident occurred in July 1991; data collected 3-4 months after | Ongoing |
| Carroll et al. (2010) [104] | Insurance claims | The authors describe disputes with insurance companies, loss adjusters, flood restoration companies, building contractors and various agencies. | Floods occurred in January 2005; data collected 10-13 months after the floods | Ongoing |
| Cottar et al. (2021) [128] | Assistance | In April 2017 the Quebec government announced emergency funding for ten municipalities for damages not covered by insurance. Starting in May 2017, disaster relief agencies such as the Red Cross became involved.  For the 2019 floods, in mid-April the Quebec Premier François Legault announced the plan to compensate homeowners with flooded properties up to $100,000 as well as the offer to buy out their homes at a maximum cost of $200,000. A total of $25.9million was paid out in financial aid throughout the province. Eligible residents could receive up to $250,000 but attached to this payment was the condition that they rebuild in place or move elsewhere on their own property but could not retreat. The $250,000 cap was seen as insufficient. | Floods occurred in April-May 2017 and April-June 2019; data collected October 2019-March 2020 | Not reported |
| Croüail et al. (2020) [132] | Assistance | Each evacuee received compensation of about €70,000 at the end of 7 years. Evacuees who lived in a place where the evacuation order had not been lifted could receive a lump sum of approximately €100,000. Property damages were also compensated, with the amount of compensation depending on the location of the property and duration of forced evacuation. Additional compensation was paid for job loss, income loss or extra works for implementing disaster countermeasures. | Accident occurred in March 2011; data collected between 2015-2017 | Some completed, some ongoing |
| De la Hoz et al. (2008) [113] | Assistance | New York State Workers’ Compensation system offered coverage of work-related medical treatment and wage replacement for ill workers. Several other public and private programmes were set up to compensate former World Trade Center workers for their injuries, illnesses and disabilities. | Incident occurred September 2001; participants were evaluated during 2004 | Ongoing |
| Dixon & Kaganoff Stern (2004) [37] | Compensation system as a whole – insurance, tort system, government programmes, charity | The authors captured $38.1billion in expenditures – payments by insurers accounted for more than 50%, and payments by government programmes accounted for most of the rest. Charitable contributions constituted only a modest share. No payments had been made through the tort system at the time of data collection although tort cases were being pursued.  Almost $10.6billion had gone to those seriously injured or the families of those killed. Almost $1.9billion had gone to emergency responders. | Attacks occurred in September 2001; data collected Fall 2002 | Ongoing |
| Edgeley & Paveglio (2017) [112] | Assistance | Local officials received Public Assistance totalling $2.35million to reestablish local infrastructure but requests for Individual Assistance for impacted residents were denied. State government officials unsuccessfully appealed this decision. | Fire occurred July-August 2014; data collected summer 2015 | Completed |
| Eriksen & de Vet (2021) [127] | Litigation; insurance claims | Nearly all participants had joined liability and compensation claims against Endeavour Energy, the Australian Defence Force or the NSW government. Most did receive some form of financial compensation (but no apology in most cases). Nearly all were disappointed with the outcome because of the time and energy invested.  Residents lodged 697 residential building claims and 538 contents claims contributing to over $160million AU in domestic insurance pay-outs. 82% of those who suffered a total loss of their homes were under-insured. | Bushfires occurred in October 2013; data collected late 2017 and early 2018 | Completed |
| Eyles et al. (1993) [119] | Assistance (compensation claims) | The Ministry of Environment processed small claims for out-of-pocket expenses caused by the evacuation and minor damage claims more quickly than major claims. Negotiations resolved in Summer 1991. | Fire occurred in February 1990; data collected May 1990 | Completed |
| FitzGerald et al. (2019) [140] | Assistance | Insurance and compensation arrangements were coordinated by the local council and the Queensland Government. | Flooding occurred in November 2010-January 2011; data collected January 2017 | Not reported |
| Flagg (2017) [144] | Litigation | Less than 8% of participants were pursuing litigation. Of these, 54.3% were pursuing litigation, either individually or through a class action suit, against the US Army Corps of Engineers.  Many other participants had filed home insurance claims. | Hurricane occurred in August 2005; data collected in 2008 | Ongoing |
| Flocks & Davies (2014) [36] | Claims facilities implemented to alleviate trauma on affected communities caused by prolonged litigation | BP was identified as the ‘responsible party’ and charged with establishing a claims process. The company began paying emergency compensation by May 2010, and between May-August 2010 they paid out $359,619,857. In June 2010, a $20billion compensation fund over four years was established as a way of victims being able to bypass potential legal logjams and distribute funds more quickly and efficiently. BP distributed up to $5000 to individuals with ‘straightforward cases’ within a month.  The authors also note errors that negatively affected approximately 7,300 claimants such as overpayment and erroneous denials of payment to 2,600 claimants. | Oil spill occurred in April 2010; data collected November 2011-January 2013 | Ongoing |
| Forster et al. (2022) [133] | Assistance | Participants remarked on lack of appropriate compensation from government sources. One government intervention provided farmers with a stipend. | Hurricane occurred in September 2017; data collected October 2019 | Not reported |
| Frerks & Klem (2005) [120] | Government compensation scheme | The government introduced a standard compensation scheme for tsunami survivors in January 2005, stating that relief assistance would be provided to survivors without taking into consideration income levels; that they would meet any funeral expenses required; and that families could be provided with money for the purchase of kitchen utensils. Only those who had lost a house or relatives were eligible for support. Over five weeks after the tsunami, only 30% of the eligible 960,000 people had received state compensation. Aid also came from multiple external organisations. | Tsunami occurred December 2004; data collected February 2005 | Ongoing |
| Gill et al. (2016) [100] | Litigation | The State of Alaska and US Government negotiated a settlement with Exxon for approximately $900million in 1991. The class action involved almost 33,000 plaintiffs and more than a third of households in Cordova had a member involved in litigation. In 1994 a jury awarded compensatory damages of $287million and punitive damages of $5billion, a decision which Exxon (the responsible party) appealed. The case went back and forth between the Ninth Circuit Court of Appeals and Federal District Court for 14 years before it was taken up by the US Supreme Court, who ruled in June 2008 that Exxon was liable but reduced the punitive damage award to $507million. In late 2008, almost 20 years after the oil spill, damage awards began to be disbursed and most had received them by December 2009. | Oil spill occurred in March 1989; data collected 1991, 1992, 1995, 1997, 2000, 2001, 2006, 2009, 2013 | Completed |
| Green et al. (1990) [23] | Litigation | 128 survivors of the dam collapse participated in a lawsuit against the coal company that built the collapsed dam; this lawsuit included claims of psychic impairment, property damage and wrongful death. Litigants were compared to 78 non-litigant survivors and a non-exposed sample. The lawsuit was settled out of court in Summer 1974 and awards for psychological damages were made to the plaintiffs. | Incident occurred in February 1972; data collected February-July 1986 | Completed – lawsuit settled 12 years before data collection |
| Hadfield (2008) [139] | Victim Compensation Fund and litigation | Those who lost family members in the 9/11 attacks were offered a quick, guaranteed payment from the Victim Compensation Fund (VCF) comparable to what they might receive from litigation. Ultimately, fewer than 100 lawsuits were filed and the VCF paid out 5,500 claims with an average of $1.2million each ($2million for the people who lost a family member; $300,000 for the injured). The study sample also included 10 plaintiffs who chose to file a lawsuit rather than accept money from the VCF. | Terrorist attacks occurred in September 2001; interview data collected in early 2004, survey data collected October 2005-June 2006, additional interviews conducted May-June 2006 | Not reported |
| He et al. (2021) [121] | Assistance | Between June 2011-July 2015 he New Zealand government offered two types of financial assistance to homeowners who held full property insurance in ‘red zones’ (areas deemed unsuitable for inhabitation). First, they offered to purchase the house and land at the 2007-08 rateable value and take over all related insurance claims; second, they offered to purchase land only, leaving property owners to deal with their own insurance companies regarding damage. By December 2015, 96% had accepted one of the two offers. | Earthquakes occurred in September 2010 and February 2011; data collected October-November 2017 | Completed |
| Heyse (2001) [103] | Not reported – ‘flood compensation’ | Not reported. | Floods began in July 1997; data collected over a two-month period in Spring 2000 | Not reported |
| Jones et al. (2018) [129] | Compensation | Daycare fire: All affected parents received a one-time monetary compensation from the government. Injured children were guaranteed lifetime medical benefits. Mothers of injured children were provided a lifelong pension.  Mine spill: Mine spill fund of approximately 150million dollars was developed using funds from Grupo México, to be managed by the government. The fund was used to compensate those who had lost income due to the spill. Compensation was based on the number of cattle an individual lost and amount of land taken out of production. There was also a programme to compensate those whose businesses had been negatively affected and homeowners who had lost access to water. | Fire occurred in June 2009; data collected January-May 2010 and January-April 2011  Copper mine spill occurred in August 2014; data collected October 2014 | Not reported |
| Joseph & Jaswal (2014) [108] | Assistance | Depending on the nature of the injury, total amount received from the central and state government ranged up to $10,141 (USD). On average, the injured received $1,749 USD. Variation in amount received was high. *Ex gratia* payments for the families of the deceased ranged from $241 USD - $95,128 USD. | Incident occurred November 2008; data collected two years later | Completed |
| Kammer-bauer & Wamsler (2017) [110] | Assistance | Government compensation was provided to low-income and uninsured homeowners, and supplemented by private donations from charitable funds. | Floods began May 2013; data collected during 2013-2016 | Not reported |
| Kaniasty (2012) [98] | Assistance | Disaster aid – not described. | Floods occurred in July 1997; data collected July-August 1998 and again 20 months post-flood | Not reported |
| Komlósi et al. (2015) [105] | Assistance | Donations were offered from both within the country and abroad. The Hungarian Remediation Fund was established by the state to manage the donations; use of donations was placed under governmental control. | Incident occurred in October 2010; unclear when data collected | Not reported |
| Marshall et al. (2004) [99] | Litigation | More than five years after the oil spill, following what the authors describe as a “very aggressive discovery phase during which Exxon’s attorneys focused on reducing damage claims and deposing thousands of plaintiffs” (p.293) the trial itself started in May 1994. Following an “aggressive and intrusive” legal strategy (p.294), many plaintiffs had their damage claims dismissed. The trial ended in September 1994 with the jury awarding a $5.2billion punitive damage verdict for the 40,000+ plaintiffs involved. Exxon’s attorneys filed repeated motions to overturn this verdict. In September 1996, the award was subject to interest payments. Exxon continued to appeal for a new trial and reduction of the punitive damage award from the original verdict, but in 2001 the Federal Court for the District of Alaska ruled that the damage award was justified. At the time of data collection, the authors claimed that Exxon planned to take the case to the US Supreme Court. | Oil spill occurred in March 1989; data collected 1991, 1992, 1995, 1997 and 2000 | Ongoing |
| Mayer et al. (2015) [90] | Compensation scheme; litigation | BP implemented a compensation process immediately after the oil spill, and began accepting claims just days after the oil spill began. From April-August 2010, BP received 154,000 claims and awarded $399million in damages, but claims data suggest that less than a third of the total claims submitted were actually fulfilled. Unresolved and future claims were handed off to the Gulf Coast Claims Facility which operated for 18 months, distributing $6.2billion to over 220,000 claimants. BP also provided limited employment opportunities in the Gulf region.  Over 110,000 private claimants also chose to participate in the class action lawsuit against BP. | Oil spill occurred in April 2010; focus groups conducted November 2011-April 2012; interview recruitment began December 2011 | Ongoing |
| Min et al. (2010) [97] | Not specified; presumed to be assistance | Not reported. | Flood occurred in July 2006; data collected 2 months pre-flood and 18 and 24 months after the flood | Not reported |
| Morris et al. (2013) [141] | Not specified; presumed to be both assistance and litigation | Compensation was administered by British Petroleum, the Gulf Coast Claims Facility, or the US Court System. | Oil spill occurred in April 2010; data collected in 2011 and 2012 | Not reported |
| Muzamil et al. (2021) [122] | Assistance | Monetary compensation provided by the government. | Floods began July 2010; data collected over 9 months in 2017 and 2019 | Not reported |
| Ng (2016) [126] | Assistance | The local government established a disaster management office and provided monetary compensation of approximately $150 USD. | Floods began July 2011; unclear when data collected | Not reported |
| Palinkas et al. (1993) [142] | Assistance | Participants received compensation for the use of boats and equipment owned by local residents and Exxon provided monetary compensation for participation in clean-up activities. | Oil spill occurred in March 1989; data collected March 1990-May 1990 | Not reported |
| Patel (2022) [68] | Assistance | The government provided grants of £5000. | Floods occurred in December 2013-January 2014; data collected April-June 2015 | Not reported |
| Picou (2009) [87] | Litigation | Litigants filed hundreds of lawsuits. In 2006 the Ninth Circuit cut the punitive damage award to $2.5billion and Exxon’s attorneys announced that they would appeal their case to the US Supreme Court. Protracted litigation concluded with a 2008 US Supreme Court decision ruling that Exxon was responsible for the oil spill but further reduced punitive damages from $2.5billion to $507million USD. | Oil spill occurred in March 1989; structured interview data collected in 1989, 1991, 1992, 1995, 2000, 2006 and 2009; qualitative interview data collected in 1995, 2000, 2006 and 2009 | Ongoing throughout data collection but completed by the end of data collection |
| Picou & Gill (2000) [123] | Litigation | Litigants filed hundreds of lawsuits and at the time of publication, legal proceedings were predicted to continue well into the next century. | Oil spill occurred in March 1989; data collected over an 8-year period, dates not reported | Ongoing |
| Picou & Hudson (2010) [91] | Insurance claims and grants | The resolution of insurance claims and payments of federal home owner grants progressed at a “painstakingly slow pace” (p.516). Of the 810 participants, around 9% reported their insurance claims were denied and more than 40% of those who had filed a claim with the federally funded relief programme had claims that were either denied or still pending. | Hurricane occurred in August 2005; data collected April-May 2008 | Ongoing |
| Picou & Martin (2007) [87] | Litigation | Approximately 38% of participants were pursuing legal action against Exxon. | Oil spill occurred in March 1989; data collected in November-December 2000 and Spring 2006 | Ongoing |
| Picou et al. (2004) [93] | Litigation | The Exxon Valdez oil spill resulted in hundreds of civil and criminal lawsuits which at the time of publication were predicted to continue well into the future. As of 2003, no substantive damage payments had been made despite a $5.3billion jury verdict in September 1994. | Oil spill occurred in March 1989; data collected July 1992 | Ongoing |
| Reid (2013) [124] | Assistance | The Federal Emergency Management Agency (FEMA) provided rental assistance programmes for displaced survivors, paying their rent directly to their landlords. The end date for this programme changed many times, sometimes being extended just days before it was scheduled to end. Eligible survivors had to reapply after 18 months but many did not know this, or know how to reapply. Additionally, after reapplying, FEMA sent cheques for funds to be used for three months of rent which the tenants had to pay and then submit rental receipts to FEMA; however, many were not aware of this. | Hurricane occurred in August 2005; data collected September 2005-August 2006 (first interviews; unclear when later interviews took place) | Ongoing |
| Ritchie (2012) [106] | Litigation | Protracted litigation concluded with a 2008 US Supreme Court decision ruling that Exxon was responsible for the oil spill but reducing punitive damages from $2.5billion to $507million USD. | Oil spill occurred in March 1989; data collected August 2002-June 2003 | Ongoing |
| Ritchie & Long (2021) [94] | Litigation | More than 63 spill-related lawsuits were filed, while the Tennessee Valley Authority (TVA) contended that it could not be held liable as it was immune from lawsuits due to being part of the federal government. In March 2010 the US District Court ruled against the TVA, and the following year, the TVA filed a second motion to dismiss the claims. The Judge ruled that plaintiffs could move forward with their cases, but they allowed TVA’s request to disallow personal injury and distress claims. The trial concluded in October 2011 and the following summer the Judge ruled against TVA, who then contested again. The final ruling, in August 2014, was that $27.8 million must be paid by TVA to more than 800 people. | Incident occurred in December 2008; data collected in 2014 (date not reported) | Not reported |
| Ritchie et al. (2013) [138] | Litigation | Protracted litigation concluded with a 2008 US Supreme Court decision ruling that Exxon was responsible for the oil spill but reducing punitive damages from $2.5billion to $507million USD. | Oil spill occurred in March 1989; data collected between 2002-2010 | Ongoing throughout data collection but completed by the end of data collection |
| Ritchie et al. (2018) [74] | Litigation | The oil spill generated many claims, settlement and litigation processes. Beyond lawsuits associated with deaths and injuries as a result of the initial explosion, the US Department of Justice filed criminal and civil suits against BP, Transocean and Halliburton. In January 2013, BP and the Justice Department settled the case for $4billion in penalties. As the responsible party, BP was also required to institute a claims process to address economic losses for individuals and businesses. In June 2010, BP were instructed by the US federal government to pay $20billion in damage claims and the initial wave of claims was almost 144,000. Between May and August 2010, BP paid out more than $395million.  In August 2010 the Gulf Coast Claims Facility was established, which disbursed $6.3billion to almost 225,000 claimants over 16 months.  Class action litigation lasted for more than twenty years, going through a series of appeals after the original 1994 jury decision. In 2008 the Supreme Court ruling cut punitive damages by 90%. | Oil spill began in April 2010; data collected in 2013 | Completed |
| Sapat et al. (2023) [111] | Assistance | Winter Storm Uri was declared a disaster which triggered a standard disaster assistance application process through FEMA. For damage caused to homes, cars and possessions, individuals and households could apply for assistance under federal programmes such as the Individual and Household Program and the Small Business Administration program. Other sources of assistance included private insurance, non-profit organisations, the National Flood Insurance Program, the Red Cross and the Department of Housing and Urban Development. | Storm occurred in February 2021; data collected May 2021 | Not reported – assumed to be completed |
| Scott et al. (1995) [102] | Litigation | Over 800 individuals made claims for financial compensation from Pan Am’s insurers. | Bombing occurred in December 1988; data collected a mean of 13 months after disaster (T1) and a mean of 36 months post-disaster (T2) | Completed |
| Simington (2023) [115] | Assistance | After the hurricanes, Marion County was allotted approximately $17million in Community Development Block Grant-Disaster Recovery funds. Residents were eligible to apply for FEMA’s Individual and Household Program. | Floods occurred in 2015, Hurricane Matthew occurred in 2016 and Hurricane Florence occurred in 2018; data collected summer 2019 | Not reported |
| Sodeyama et al. (2022) [95] | Assumed to be assistance | Where evacuation was ordered, the government provided compensation for evacuation (to compensate for psychological impacts and impairment of daily living), evacuation costs (e.g., travel, accommodation) and loss of income. In the absence of evacuation orders, compensation was either not provided at all or was inadequate. Amount of compensation differed depending on whether an area was classified as a difficult-to-return area or a restricted-residence area. | Incident occurred in March 2011; data collected October-December 2016 | Not reported – assumed to be completed |
| Sou et al. (2021) [117] | Assistance | FEMA rapidly assessed household needs of financial aid for recovery and reconstruction and provided funds to eligible cases. Eligibility was determined via an onsite assessment of structural damage, a detailed application by the household, and provision of proof of legal ownership of the property. The approval process took 2-6 months. | Hurricane occurred in September 2017; data collected through five visits between October 2017-September 2018 | Ongoing |
| Sterett (2012) [116] | Assistance | Housing assistance payments were provided by FEMA and depended on people having evacuated. People transitioned from emergency assistance which everyone from flood-damaged areas could claim to individual assistance which required recertification for eligibility every three months. | Hurricane occurred in August 2005; data collected within 6 months of the hurricane (first wave) and six months after the first interview (second wave) | Ongoing |
| Sungur & Kaya (2001) [101] | Litigation | Court hearings held 18 months after the disaster. Compensation not described, but the outcome was reportedly not ideal for the participants. | Incident occurred in July 1993; data collected 45 days after the event, 6 months after the event, 12 months after the event and 18 months after the event | Ongoing throughout study and then completed at end of study |
| Talbot (2011) [109] | Assistance | 8/12 participants were recipients of public money over the course of the 3 years since the attacks, including money from the Victim’s Compensation Fund as well as various charities. | Terrorist attacks occurred September 2001; recruitment began October 2004 | Not reported |
| Tsujiuchi (2021) [92] | Reparations | Reparations for psychological distress were ¥14,500,000 for those who lived in hazardous areas they could not return to; ¥4,800,000 for those who lived in areas scheduled for reopening in the next few years; ¥2,150,000 for under-18s and ¥1,800,000 for adults evacuated from areas declared uninhabitable in 2011 but reopened at the time of data collection; for those who were voluntary evacuees from areas declared not hazardous, or those who were living in temporary shelters due to the tsunami rather than the nuclear disaster, children and pregnant women received ¥520,000 and others received ¥120,000. Participants indicated it was difficult to identify when their real estate would really be compensated for by the government or the Tokyo Electronic Power Company. | Incident occurred in March 2011; data collected in February-March 2015 and compared with yearly data collected between February-March 2012-2014 | Not reported, but the suggestion that participants were unclear when their real estate would be compensated for suggests ongoing |
| Tsujiuchi et al. (2016) [96] | Not reported | Not reported. | Incident occurred in March 2011; data collected March-April 2012 | Ongoing |
| Van der Geest & Schindler (2016) [137] | Assistance | Participants received relief from organisations or the government. Some received monetary compensation of 40,000 rupees per deceased household member while others received in-kind aid. | Landslide occurred in August 2014; date of data collection not reported | Not reported |
| Van Truong et al. (2021) [125] | Remedial compensation | The Formosa Ha Tinh Steel Corporation settled with the Vietnamese government for a remedial compensation package of $500million USD. 65% of these funds were eventually transferred to impacted coastal households. Compensation differed between fishing households, fish farming households and coastal service households. Funds were released 1-2 years after the disaster. | Incident occurred in April 2016; data collected August-December 2018 | Completed |
| Won et al. (2019) [114] | Compensation fund | Samsung Heavy Industry offered $88million which was refused by residents and eventually agreed to be $318million; there were five years of disputes and another five years for the funds to be delivered due to additional conflicts over distribution and which association would manage the fund.  Initial payments were distributed on a graded payment system according to where people lived and the type of job they had, which led to intense complaints. | Incident occurred in December 2007; data collected 10 years later | Ongoing |
