# Supplementary material for: Psychosocial impacts of post-disaster compensation processes: narrative systematic review
Source: BMC Psychol. 2024 Oct 7;12:539. doi: 10.1186/s40359-024-02025-9 (PMC11460075; doi:10.1186/s40359-024-02025-9)
Supplement: Supplementary file 3 — Supplementary Material 3: Additional Table 3. Caption:A typology of compensation impacts [file 40359_2024_2025_MOESM3_ESM.docx]

**Additional Table 3. A typology of compensation impacts on mental health and individual/community wellbeing**

| **Category** | **Examples from the literature** |
| --- | --- |
| Statistical Associations  (Mixed findings) | Abnormal mood [89]  Stress: Intrusive [74, 86, 87, 89, 93, 94] or avoidant [74, 94]  Depression [86, 87, 88, 90, 95]  PTSD [88, 92, 96]  Generalised anxiety disorder [23]  Recurrent unpleasant memories [93]  Suicidal ideation [95]  Poorer social psychological wellbeing [98] (dissatisfaction with the compensation process)  Better physical and mental health [97] (satisfaction with the compensation process) |
| Mental health and wellbeing: Longitudinal | Stress [86, 99, 100, 101]  PTSD [101, 102, 103] |
| Mental health and wellbeing: Compensation process - related | Stress:  Agencies involved in pay-out process [104]  Liability determination process [105]  Compensation process (more generally) [46, 47]  Compensation process as or more distressing than the disaster, itself [46, 74, 106]  Re-traumatising [106]  Seeking compensation as a means of coping [109] |
| Mental health and wellbeing: Emotions | Disappointment [107]  Anger [108]  Exhaustion [106]  Feeling powerless or defeated [46, 106]  Humiliation [105]  Distress [108]  Pessimism [106]  Stress [105, 109]  Feeling worried [46]  Claims process perceived as demanding on emotional resources [46, 106]  Sleep problems and physical health complaints [46] |
| Challenges of the claims process: | Complexity and volume of forms [36, 37, 46, 68, 106, 109-112]  Difficulties answering specific questions [68, 46]  Difficulties providing necessary documentation [46, 107, 108 – 111, 113-117]  Length of the process [36, 37, 68, 74, 88, 92, 104, 106, 108, 109, 115, 117-125]:  Living in unsuitable accommodation [46, 117, 119]  No income [36, 125]  Substantial demands on financial resources [46, 114]  Unable to comes to terms with grief [109] |
| Procedural (in)justices: Lack of communication and information | Lack of or poor-quality communication and transparency [36, 37, 46, 68, 90, 111-115, 117, 120, 124-127]  Misinformation or conflicting information provided [36, 46, 104, 105, 112, 116, 121]  Lack of eligibility [37, 92, 112, 113, 115, 116, 120, 121, 124, 128, 129]  Disproportionate disadvantages to some groups [37, 113, 115, 117, 121, 124]  Within process challenges and disagreements (e.g. valuation of homes) [105, 110, 119, 121, 127]  Lack of inter-agency cooperation [37, 120, 128, 130]  Lack of understanding communities [36, 112, 120, 130-132]  Insufficient payment [36, 68, 86, 108, 112, 114, 122, 126-128, 133] |
| Interpersonal (in)justice: | Corruption and politicisation of compensation process (non-litigation compensation) [108, 118, 120, 122, 126, 129, 130, 133-137]  Perceived fairness of disaster assistance distribution [134, 135]  Loss of trust in authorities and officials [36, 68, 93, 103, 108, 123, 125, 126, 131, 133, 138] |
| Impact on communities: | Unequal compensation [36, 37, 90, 92, 96, 109, 112, 114, 115, 117-120, 125, 132, 133, 140-142]  Seemingly arbitrary decisions [36, 90, 114, 120, 131, 133, 141]  Perceived lack of ‘deservingness’ and fraudulent claims [36, 37, 74, 90, 108, 114, 119, 122, 131, 141]  Perception that others view own compensation as dishonest [102, 109]  Envy and jealousy towards others who received more [90, 114, 119, 120, 130, 132, 141]  Being the object of others’ envy because of compensation received [90, 92, 102, 130, 132]  Community relationships disintegrating or fragmenting [36, 74, 90, 92, 93, 98, 114, 120, 123, 125, 129, 130, 131 132, 141, 143, 144]  Family relationships [108, 114, 119, 121, 144]  Sudden influx of money disrupting communities [36, 90, 116]  Negative impacts on businesses [90]  Housing impacts [128, 131, 145] |
| Other challenges and barriers: | Language barriers [37, 113]  Tax on damages awarded [86]  Feeling rushed into decisions about the future [105, 127]  Fear of deportation [37]  Feeling discouraged from applying [115]  Grief making it too challenging to engage with compensation processes [37] |
| Positive findings: | Gratitude for formal assistance [109, 118, 121, 137]  Help in restarting, recovering, or resuming livelihood activities [118, 121, 125, 128, 137]  Keeping businesses open [36]  Straightforward compensation process (very few studies) [36, 121, 127]  Receiving or giving help in navigating the compensation process [110, 115, 122]  Strengthening community bonds [126, 133, 135] |
