# Supplementary material for: Psychosocial impacts of post-disaster compensation processes: narrative systematic review
Source: BMC Psychol. 2024 Oct 7;12:539. doi: 10.1186/s40359-024-02025-9 (PMC11460075; doi:10.1186/s40359-024-02025-9)
Supplement: Supplementary file 4 — Supplementary Material 4: Additional Table 4. Caption:Recommendations based on the review’s findings [file 40359_2024_2025_MOESM4_ESM.docx]

**Additional Table 4. Recommendations based on the review’s findings**

| **Topic** | **Recommendation** |
| --- | --- |
| Pre-disaster emergency planning | - Pre-disaster, ensure that emergency preparedness and response plans include details about potential compensation funds and disaster assistance, including how eligibility criteria will be determined; which agencies will be involved; how information will be disseminated; how to identify those particularly in need; how to eliminate fraud; and how payments should be made. At the same time, be flexible and acknowledge that pre-disaster decisions may change depending on the nature of the disaster and its aftermath. |
| Communication | - Improve communication to ensure all eligible recipients of compensation / assistance are aware of and able to access support. This should include active outreach to marginalised groups. - Ensure communication throughout the process is clear, consistent, credible and accessible [150]. If information is not available, be upfront about that and update people as soon as possible [157]. - Develop and disseminate a document, outlining in clear and simple terms the criteria for compensation eligibility. Justify all eligibility criteria. - Be transparent about how compensation decisions are made. - Provide regular updates throughout the process. - Collaborate with the media to ensure they do not fuel public resentment [158]. |
| Mental health | - Develop clinical intervention programmes to better understand and mitigate the psychosocial stress associated with compensation processes and ensure that clinicians treating patients involved in compensation claims understand the mental health impacts of them. Ideally, communities would be able to offer free mental health services scaled to community needs, recognising that compensation inequities can be a psychosocial stressor and can potentially retraumatise people. - Offer stepped care, with communities supported to look after themselves; if needed, also offer free, accessible and timely mental health services scaled to community needs, recognising that perceived compensation inequities may be a psychosocial stressor. |
| Inclusivity and consideration of community-specific needs | - Consider how to include vulnerable groups such as immigrants and undocumented workers in the compensation process. Be sensitive to gender, race and socio-cultural norms [120]. - Involve affected communities in shaping decision-making, providing local information to assessors, plans for distribution of compensation. - Establish the needs of the affected community, taking into account disproportionately vulnerable groups [136]. Ensure the values and norms of individual communities are understood [112, 157]. - Consider, based on local knowledge and with input from affected individuals themselves, whether they would benefit from one-off lump sum direct payments or whether this might be disruptive to communities. |
| Fairness | - Ensure those in charge of compensation processes are efficient, organised and fair [36]. - Ensure the procedures for assessing losses and determining compensation are objective, systematic and as fair as possible. - Consider timeliness and fairness of allocation. - Use external agencies to monitor compensation distribution, ensuring it is fair and not used as a means of political control [90, 136]. - Ensure there are processes in place to identify and eliminate fraud [150]. - Decrease the adversarial nature of litigation by utilising processes such as mediation and arbitration by court-appointed experts. |
| Directions for future research | - Fund research on interventions to improve community resilience when ‘perfect compensation equity’ is not feasible. - More research is warranted on interventions to reconcile divided communities post-disaster. |
