# Supplementary material for: Psychosocial impacts of post-disaster compensation processes: narrative systematic review
Source: BMC Psychol. 2024 Oct 7;12:539. doi: 10.1186/s40359-024-02025-9 (PMC11460075; doi:10.1186/s40359-024-02025-9)
Supplement: Supplementary file 6 — Supplementary Material 6: Appendix 2. This is the search strategy [file 40359_2024_2025_MOESM6_ESM.docx]

**Appendix 2: Example search strategy; PsycInfo**

1. compensation

2. compensatory pay*

3. payout*

4. compensatory claim*

5. "claims process*"

6. "pay damages"

7. "damage pay"

8. litigation

9. afcs

10. “heads of claim”

11. compensatory award*

12. “quantum of damages”

13. “public liability”

14. “disaster aid”

15. “disaster insurance”

16. “monetary relief”

17. “financial relief”

18. “financial assistance”

19. “monetary assistance”

20. 1 or 2 or 3 or 4 or 5 or 6 or 7 or 8 or 9 or 10 or 11 or 12 or 14 or 15 or 16 or 17 or 18 or 19

21. disaster*

22. emergenc*

23. “oil spill*”

24. “chemical spill*”

25. pandemic*

26. earthquake*

27. hurricane*

28. "industrial accident*"

29. "volcanic eruption"

30. landslide*

31. typhoon*

32. tornado*

33. tidal wave*

34. storm*

35. terroris*

36. tsunami*

37. flood*

38. fire*

39. cyclone*

40. explosion*

41. avalanche*

42. blizzard*

43. wildfire*

44. bushfire*

45. 21 or 22 or 23 or 24 or 25 or 26 or 27 or 28 or 29 or 30 or 31 or 32 or 33 or 34 or 35 or 36 or 37 or 38 or 39 or 40 or 41 or 42 or 43 or 44

46. mental health

47. wellbeing

48. well-being

49. psychological

50. psychosocial

51. psychiatric

52. "adjustment disorder*"

53. "mental disorder"

54. depression

55. anxiety

56. panic

57. ptsd

58. trauma*

59. stress*

60. post-traumatic

61. distress*

62. resilien*

63. mood*

64. emotion*

65. quality of life

66. life satisfaction

67. coping

68. sleep

69. corrosion

70. corroded

71. inequ*

72. competiti*

73. jealous*

74. fairness

75. unfair*

76. justice

77. injustice*

78. “social impact*”

79. “community social relations*”

80. “community relations*”

81. envy

82. envious

83. “social comparison”

84. occupational

85. colleague*

86. 46 or 47 or 48 or 49 or 50 or 51 or 52 or 53 or 54 or 55 or 56 or 57 or 58 or 59 or 60 or 61 or 62 or 63 or 64 or 65 or 66 or 67 or 68 or 69 or 70 or 71 or 72 or 73 or 74 or 75 or 76 or 77 or 78 or 79 or 80 or 81 or 82 or 83 or 84 or 85

87. 20 and 45 and 86
